# Emerging roles of interactions between ncRNAs and other epigenetic modifications in breast cancer

**DOI:** 10.3389/fonc.2023.1264090

**Published:** 2023-10-12

**Authors:** Junyuan Xie, Li Gan, Bingjian Xue, Xinxing Wang, Xinhong Pei

**Affiliations:** Department of Breast Surgery, The First Affiliated Hospital of Zhengzhou University, Zhengzhou, China

**Keywords:** ncRNAs, breast cancer, DNA methylation, RNA methylation, histone modification

## Abstract

Up till the present moment, breast cancer is still the leading cause of cancer-related death in women worldwide. Although the treatment methods and protocols for breast cancer are constantly improving, the long-term prognosis of patients is still not optimistic due to the complex heterogeneity of the disease, multi-organ metastasis, chemotherapy and radiotherapy resistance. As a newly discovered class of non-coding RNAs, ncRNAs play an important role in various cancers. Especially in breast cancer, lncRNAs have received extensive attention and have been confirmed to regulate cancer progression through a variety of pathways. Meanwhile, the study of epigenetic modification, including DNA methylation, RNA methylation and histone modification, has developed rapidly in recent years, which has greatly promoted the attention to the important role of non-coding RNAs in breast cancer. In this review, we carefully and comprehensively describe the interactions between several major classes of epigenetic modifications and ncRNAs, as well as their different subsequent biological effects, and discuss their potential for practical clinical applications.

## Introduction

Breast cancer is the most common type of cancer in women worldwide, and both developed and developing countries are seeing increased rates of morbidity and mortality from the disease (1, 2). In China, the incidence and mortality of breast cancer rank first among female malignant tumors (3). At the same time, the lack of early clinical features and low-cost screening means breast cancer is often detected at a later stage, delaying treatment (4). Currently, the treatment of breast cancer mainly includes chemotherapy, radiotherapy, targeted therapy, immunotherapy, endocrine therapy and so on (5). However, the therapeutic effect and long-term prognosis still need to be further improved.

Epigenetic modification refers to changes in gene expression that are independent of changes in DNA sequence, mainly including DNA modification, histone modification and RNA modification (6, 7). DNA methylation is the most intensively studied epigenetic regulation mechanism (8). DNA methylation regulates growth and development, gene expression pattern and genome stability without changing DNA sequence, and abnormal expression of DNA methylation can lead to the occurrence and development of tumors (9, 10). At present, histone modification mainly includes methylation, phosphorylation, acetylation and so on (11, 12). Histone methyltransferase can directly regulate the methylation site and degree of histone, and the methylation of different sites of histone H3 and H4 is very important for transcriptional regulation of genes (13, 14). For RNA methylation modification, the current research mainly focuses on m6A modification, which accounts for more than 80% of all RNA methylation (15). m6A modifications regulate mRNA at different levels and participate in various cell life activities, such as cell cycle regulation and cell differentiation (16, 17). The above three epigenetic modifications together constitute the three major elements of basic regulation of life activities.

With the continuous maturation of high-throughput sequencing technology, more and more non-coding RNAs (ncRNAs) appear in the public eye (18). The ncRNAs closely associated with cancer mainly include miRNAs, piRNAs, circRNAs and lncRNAs (19). These ncRNAs change the abnormal expression of cancer suppressor or oncogene mainly through epigenetic modification at different levels, thus leading to the progression and occurrence of cancer (20, 21).

In this review, we focused on the current carcinogenic role of ncRNAs in the occurrence and development of breast cancer through epigenetic modification, their core regulatory mechanisms and their effects on the biology of breast cancer, hoping to provide new insights into clinical treatment and prognosis of breast cancer in the future.

## Core members involved in epigenetic modifications

**Figure 1:**
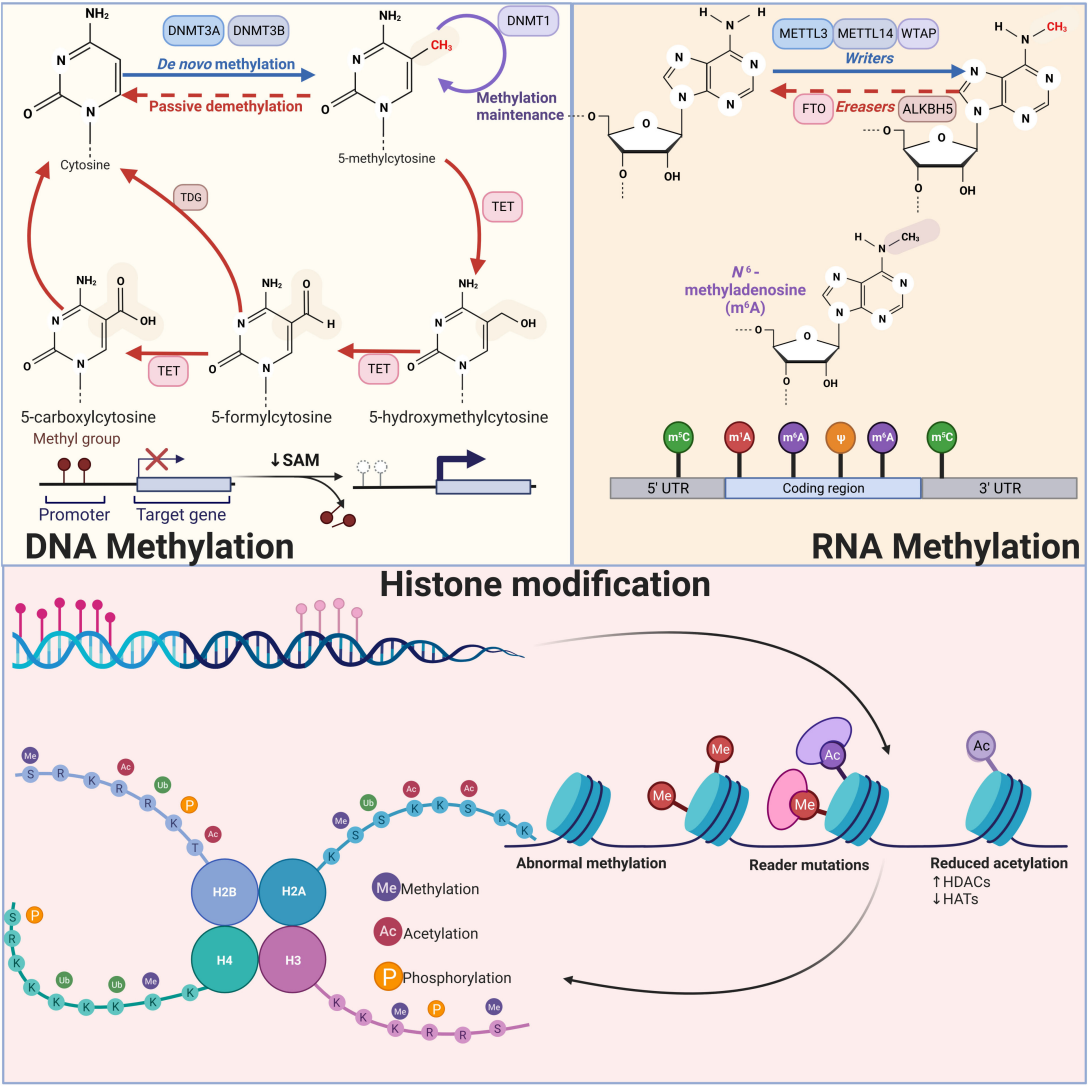
Schematic diagram of three types of epigenetic modifications: DNA methylation, RNA methylation, and histone modification DNA methylation inhibits transcription when it is near promoter regions, whereas methylation within the genome promotes gene expression. DNMT3A and NDMT3B catalyze *de novo* methylation, adding a methyl group to the fifth carbon of a cytosine, while DNMT1 is primarily responsible for maintaining DNA methylation levels. RNA methylation modifications mainly include m6A, m5C, m1A and m7G, among which m6A modification has been studied most thoroughly. In m6A modification, Writers including METTL3, METTL14 and WTAP are able to add methyl groups to the sixth N position of the adenylate, while Erasers are able to remove the methylation, reversing the function of Writers. Histone modification mainly includes methylation, acetylation, and phosphorylation. Histone methylation occurs mainly in arginine and lysine residues and is a reversible process that can be finely controlled by various demethylases (KDM) and methyltransferases (KMT). Histone acetylation is mainly regulated by acetyltransferases, which mainly add acetyl groups to lysine residues of histones. A number of kinases and phosphatases control the histone phosphorylation process, primarily affecting serine, tyrosine, and threonine residues in the histone tail.

In the past few decades, more and more evidence has been found to confirm the important role of epigenetic modification in the regulation of various human cancers (22, 23). In addition, epigenetic modification involving emerging ncRNAs has also attracted great attention of researchers (24, 25). In breast cancer, epigenetic modification involving ncRNAs mainly focuses on DNA methylation, RNA methylation and histone modification (12, 26, 27) (**Figure 1**). These modifications work mainly through key regulatory enzymes such as methylase, acetylase and ubiquitination enzyme (16, 28, 29).

### DNA methylation

DNA methylation, the most extensively researched epigenetic mechanism, is essential for maintaining the genome integrity and controlling mammalian gene expression (30, 31). Generally speaking, methylation near the promoter region inhibits transcription while methylation in the genome encourages gene expression (32). DNA methylation refers to the modification of methyl groups on the fifth carbon of cytosine (5-methylcytosine, 5mC), commonly found in mammalian symmetric CpG dinucleotides (33). This process is catalyzed and regulated by DNA methylation transferase (DNMT), Currently, known DNMTs involved in DNA methylation mainly include DNMT1, DNMT3A and DNMT3B (34). DNMT1 is required to maintain DNA methylation levels during cell replication (35). In early and late embryos, DNMT3A and NDMT3B, which are both extremely homogenous, catalyze *de novo* methylation (36, 37). Even though it lacks methyltransferase activity, DNMT3L aids DNMT3A/B in carrying out its job.

### RNA methylation

Epigenetic transcriptome modification (RNA modification) has become a key regulatory factor of gene expression during eukaryotic development (15). To date, more than 170 different types of chemical modifications have been identified on the RNA nuclear bases and play key roles in different biological processes (38). In breast cancer, studies on RNA modification mainly focus on m6A methylation (39, 40). Therefore, in this review, we will focus on the important role of m6A methylation in breast cancer. Writer, eraser, and reader are the three principal proteins that currently control m6A modification (41). Among the writers who promote methylation are METTL3, METTL14, WTAP, RBM15, ZC3H13, and VIRMA (42, 43). The most widely known catalyst for m6A modification, METTL3, has the capacity to bind to the methyl donor SAM and effect methyl transfer (44). It is believed that METTL14 functions as an RNA-binding platform that, along with METTL3, creates the core methyltransferase complex that catalyzes and produces the m6A alteration (45). WTAP serves as a crucial adapter protein, increasing the stability of the core methyltransferase complex in the meantime (46). For the purpose of enlisting targets of the methyltransferase complex, RBM15/15B can bind to METTL3 and WTAP. The cornerstone for the varied regulation of m6A modification is the quantity of methyltransferases (47). Erasers, such as Fat mass and obesity-associated protein (FTO) and AlkBhomolog5(ALKBH5), are demethylases capable of removing m6A modifications from RNA (48). The readers include the m6A-specific methylation proteins IGF2BP1/2/3, YTHDF1/2/3, and ELAVL1, which can recognize and bind to m6A-modified transcripts through a variety of processes, thereby regulating gene expression, mRNA splicing, mRNA structure, translation efficiency, and miRNA biogenesis (49). These three categories of regulatory proteins influence the development of cancer and the prognosis of patients by controlling many downstream molecules and signaling cascades.

### Histone modification

Chromatin is a dynamic molecule with a variety of structures, and histones are one of the major components of chromatin with a tail protruding from the nucleosome, which is susceptible to covalent modification in several places (50). It includes a variety of covalent modifications, such as acetylation, phosphorylation, methylation, ubiquitination, and sumoylation (13, 51–53). The nucleosome, which is made up of two copies of the histones H2A, H2B, H3, and H4, is the fundamental building block of chromatin. It wraps around about 147 DNA base pairs (54). At present, more efforts have been put into histone modification in two aspects, acetylation and methylation. Covalent modification of histones can alter nucleosome conformation, thereby regulating chromatin structure and gene expression (55). Histone methylation occurs mainly in arginine and lysine residues. Arginine is asymmetrically or symmetrically methylated, whereas lysine is monomethylated, dimethylated, or trimethylated (56). Histone methylation is a reversible process that can be finely controlled by various demethylases (KDM) and methyltransferases (KMT). H3K4, H3K36, and H3K79 are some of these markers that are connected to transcriptional activation, while H3K9, H3K27, and H4K20 are connected to transcriptional repression (57). At several lysine residues in the histone tail, acetyl groups can be added during histone acetylation. From a functional standpoint, we are aware that histone acetylation, particularly when localized at enhancers, promoters, and gene bodies, is substantially linked to active transcription (58). A number of malignancies have been discovered to have altered overall levels of histone acetylation, particularly H4K16ac, which has even been found to have possible predictive significance (59). Gene expression may be stimulated when hyperacetylation takes place, particularly when proto-oncogenes are involved, whereas hypoacetylation of tumor suppressors is typically localized at the promoter and takes place concurrently with DNA methylation, resulting in gene silence (58). The enzyme that adds acetyl groups to the lysine residues of histones is lysine acetyltransferase (KAT), commonly referred to as histone acetyltransferase (HAT). The opposite histone deacetylase (HDAC) is responsible for its removal. HAT and HDAC activity together are required for proper regulation of gene expression (60). Thus, given that acetylation modification is reversible, pharmacological interventions targeting HAT, HDAC, and acetyl-lysine readers have potential value for therapeutic cancer therapy. Numerous kinases and phosphatases control the process of histone phosphorylation, which mainly affects the serine, tyrosine, and threonine residues in the histone tail (61). Histone phosphorylation during mitosis upsets the equilibrium of connections between histones and DNA, resulting in unstable chromatin structure that eventually influences the development and occurrence of cancer (62).

### ncRNAs involved in epigenetic modification

Non-coding RNAs (ncRNAs) are composed of many types of RNAs that are not translated into proteins (63). Abnormal expression of ncRNAs has been found and confirmed to be closely related to clinical prognosis in many types of cancer, including breast cancer (64). ncRNAs can be divided into two main categories based on their length. Small/short ncRNAs (sncRNAs), which have a length of 200 nucleotides or less, are the first class of ncRNAs (65). They are mostly made up of microRNAs (miRNA), piwi interacting RNAs (piRNA), endogenous short interfering RNAs (endo-siRNAs), and small nucleolar RNAs (snoRNAs) (66). The second category includes ncRNAs >200 nucleotides in length and is called long ncRNAs (lncRNAs) (67). CircRNA is a recently discovered ncRNA with a covalently closed structure, and although it does not contain the 5’-3’ polar RNA polymerase II transcription or polyadenine tail, it has the same transcription efficiency as linear RNA (68). The above ncRNAs have changed the biological behavior of breast cancer cells by interacting with various epigenetic modifications, and indirectly affect the occurrence and development of cancer.

## Interactions between ncRNAs and other epigenetic modifications in breast cancer

**Figure 2:**
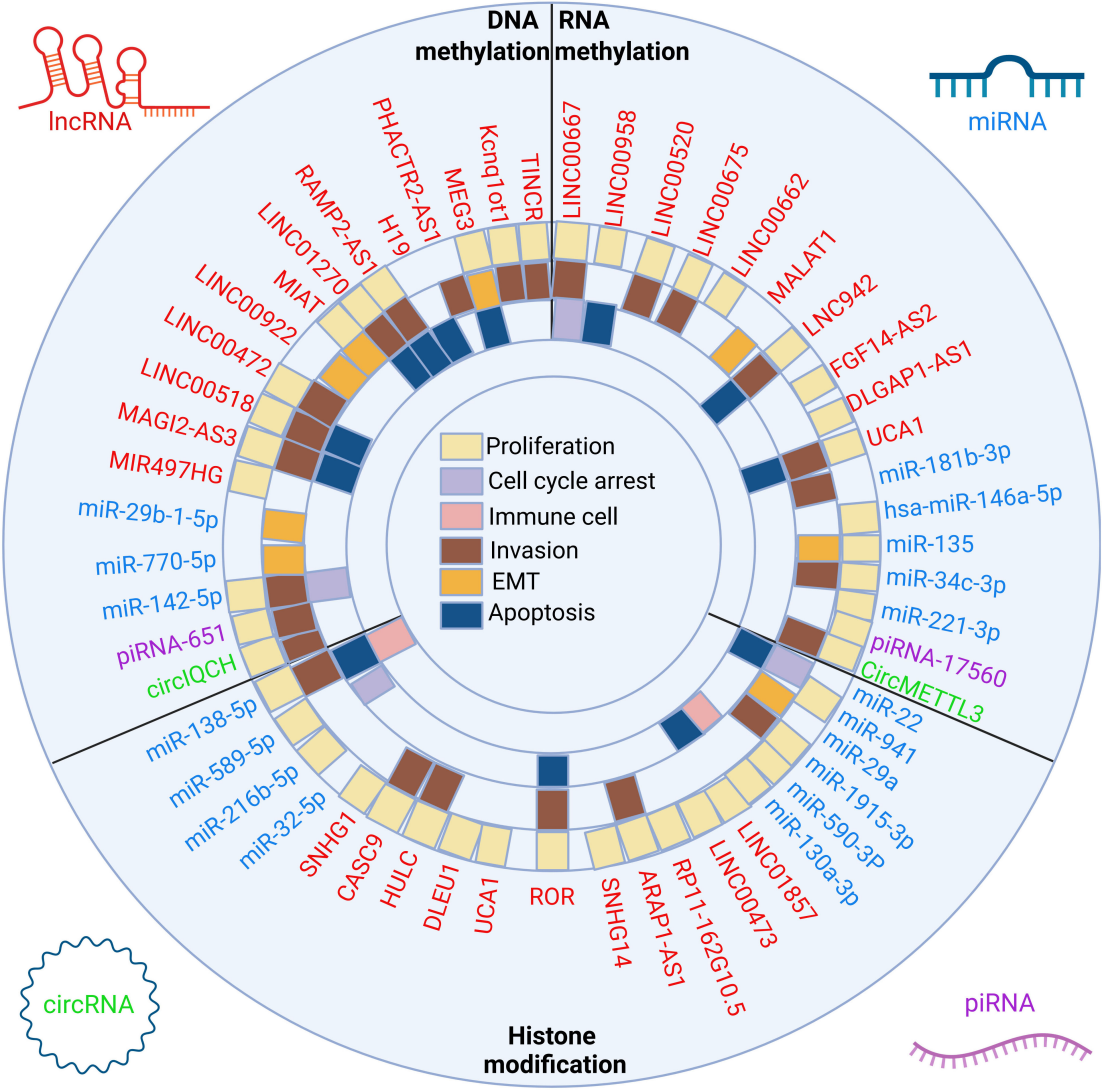
The crosstalk between ncRNAs and three classes of epigenetic modifications and biological implications. The epigenetic modifications can be divided into DNA methylation, RNA methylation and histone modification. LncRNAs, miRNAs, circRNAs and piRNAs affect the proliferation, apoptosis, cell cycle arrest, EMT, invasion and immune microenvironment of breast cancer cells by affecting the above epigenetic modifications.

As one of the special forms of epigenetic modification, ncRNAs can interact with DNA methylation, RNA methylation, and histone modification through various modifying enzymes, and regulate the expression of specific genes (69–71), thereby affecting the expression of downstream genes and regulating the progression of cancer (**Figure 2**). In this section, we mainly introduce the crosstalk mechanism between ncRNA and DNA methylation, RNA methylation and histone modification through three parts (**Table 1**).

**Table 1:** Interaction of ncRNAs with other epigenetic modifications.

Epigenetic modification	modification enzyme	ncRNAs	Initial regulator	Axis	Ref.
DNA modification	DNMT1	TINCR	lncRNA	STAT3-TINCR–EGFR	(72)
	DNMT1	TINCR	lncRNA	STAT1-TINCR-USP20-PD-L1	(73)
	DNMT1	Kcnq1ot1	lncRNA	YY1–lncRNA Kcnq1ot1–PTEN	(74)
	DNMT1	LncRNA MEG3	modification enzyme	DNMT1-MEG3-Notch1 signaling pathway	(75)
	DNMT1	LncRNA PHACTR2-AS1	modification enzyme	PAS1/PH20	(76)
	DNMT1	miR-142-5p	microRNA	MKL-1/miR-142-5p/DNMT1/maspin	(77)
	DNMT1	piRNA-651	piRNA	piR-651/PTEN	(78)
	DNMT3A	circIQCH	circRNA	circIQCH/miR-145/DNMT3A	(79)
	DNMT3A	miR-770-5p	microRNA	miR-770-5p/DNMT3A/CDH1	(80)
	DNMT3B	lncRNA H19	lncRNA	H19/SAHH/Beclin1	(81)
	DNMT3B	LncRNA MEG3	modification enzyme	UXT/MEG3/P53	(82) (83)
	DNMT1 and DNMT3B	LncRNA RAMP2-AS1	lncRNA	RAMP2-AS1/CXCL11	(84)
	DNMT1, DNMT3A, and DNMT3B	LINC01270	lncRNA	LINC01270-LAMA2-MAPK pathway	(85)
	DNMT1, DNMT3A, and DNMT3B	lncRNA MIAT	lncRNA	lncRNA MIAT-DLG3-Hippo signaling pathway	(86)
	DNMT1, DNMT3A and DNMT3B	LINC00922	lncRNA	LINC00922/NKD2/Wnt signaling pathway	(87)
	DNMT1, DNMT3A and DNMT3B	miR-29b-1-5p	microRNA	NRF2/miR‐29b‐1‐5p/AKT signaling pathway	(88)
	DNMT1, DNMT3A and DNMT3B	LINC00472	lncRNA	LINC00472/MCM6/MEK/ERK signaling pathway	(89)
	DNMT1, DNMT3A and DNMT3B	LINC00518	lncRNA	LINC00518/CDX2/Wnt signaling pathway	(90)
	/	MAGI2‐AS3	lncRNA	AKT signal pathway, Wnt signal pathway, and BAX/BCL2 apoptosis pathways	(91)
	DNMT3B and HDAC1/2	lncRNA MIR497HG	modification enzyme	MIR497HG/miR-195 and miR-497/PI3K/AKT Signaling pathway	(92)
RNA modification	METTL3	miR-34c-3p	microRNA	circMETTL3/miR-34c-3p/METTL3	(93)
	KIAA1429	LINC00667	Positive feedback loop	KIAA1429/mA/LINC00667/miR-556-5p feedback loop	(10)
	METTL3	LINC00958	modification enzyme	LINC00958/miR-378a-3p/YY1	(94)
	METTL3	LINC00520	modification enzyme	LINC00520/miR-577/POSTN/ILK/AKT/mTOR signaling pathway	(95)
	METTL3	LINC00675	modification enzyme	LINC00675/miR-513b-5p	(96)
	METTL3	LINC00662	modification enzyme	LINC00662/miR-186-5p/METTL3	(97)
	METTL3	MALAT1	modification enzyme	MALAT1/miR-26b/HMGA2	(98)
	METTL3	CircMETTL3	modification enzyme	circMETTL3/miR-31-5p/CDK1	(99)
	METTL3	miR-221-3p	modification enzyme	miR-221-3p/HIPK2/Che-1	(10)
	METTL3	miR-135	microRNA	miR-135/ZNF217/NANOG	(100)
	METTL14	LNC942	lncRNA	LNC942/METTL14/CXCR4 and CYP1B1	(101)
	METTL14	LncRNA UCA1	lncRNA	UCA1/METTL14/miR-375/SOX12	(102)
	METTL14	hsa-miR-146a-5p	modification enzyme	METTL14/hsa-miR-146a-5p	(103)
	FTO	miR-181b-3p	modification enzyme	FTO/miR-181b-3p/ARL5B	(104)
	FTO	piRNA-17560	modification enzyme	piR-17560/FTO/ZEB1	(105)
	YTHDF2	lncRNA FGF14-AS2	modification enzyme	FGF14-AS2/RUNX2/RANKL	(106)
	WTAP	lncRNA DLGAP1-AS1	modification enzyme	DLGAP1-AS1/miR-299-3p/WTAP	(107)
Histone methylation	SUV420H2	miR-29a	microRNA	miR-29a/SUV420H2/EGR1 and CTGF	(108)
		LncRNA SNHG1	lncRNA	SNHG1/miR-381	(109)
		miR-32-5p	microRNA	CUL4B/miR-32-5p/ER-α36	(110)
	SETD1A	miR-1915-3p	microRNA	miR-1915-3p/SETD1A/	(111)
	EZH2	lncRNA UCA1	lncRNA	UCA1/EZH2/P21	(112)
	KDM6B	miR-138-5p	microRNA	miR-138-5p/KDM6B/M2 polization	(113)
	MLL1	ROR	lncRNA	LncRNA ROR/MLL1/TIMP3	(114)
Histone acetylation	HDAC2	ARAP1-AS1	lncRNA	ARAP1-AS1/HDAC2/PLIN1	(115)
	HDAC3	miR-589-5p	microRNA	miR-589-5p/HDAC3	(116)
	HDAC3	miR-130a-3p	modification enzyme	HDAC3/miR-130a-3p/HMGB3	(117)
	HDAC8	miR-216b-5p	/	/	(118)
	HDAC4 and FOXP1	miR-22	microRNA	miR-22/HDAC4/p53	(119)
	CREB	LINC00473	lncRNA	LINC00473/CCND1	(120)
	/	lncRNA HULC	lncRNA	HULC/IGF1R-PI3K-AKT	(121)
	/	DLEU1	lncRNA	DLEU1/SRP4	(122)
	/	SNHG14	lncRNA	lncRNA SNHG14/PABPC1	(123)
	/	LINC01857	lncRNA	LINC01857/CREB1	(124)
	/	miR-590-3P	microRNA	miR-590-3P/P53	(125)
	/	LncRNA CASC9	lncRNA	CASC9/SOX4	(126)
	/	lnc RP11-162G10.5	/	/	(127)
Histone phosphorylation	/	miR-941	microRNA	/	(128)

### Crosstalk of ncRNAs and DNA methylation in BC

ncRNAs can not only actively recruit DNA methyltransferases to actively regulate the methylation of downstream gene promoters, but also act as a downstream target that is actively regulated by DNA methyltransferases (129).

#### Active regulation of DNA methylation by ncRNAs

As a carcinogenic factor, TINCR can promote the progression of breast cancer *in vivo* and *in vitro*. Wang et al. demonstrated that this is due to TINCR’s ability to recruit DNMT1 into the promoter of miR-503-5p, thereby inhibiting its expression through DNA methylation, and ultimately leading to high expression of downstream target STAT3. Surprisingly, further studies demonstrated that STAT3 was highly enriched at the promoter of the TINCR site and involved in transcriptional regulation of TINCR, suggesting that a complete positive feedback loop was formed between STAT3 and TINCR (72). Later, Wang et al. demonstrated that TINCR recruited DNMT1 into the promoter region of miR-199a-5p in the nucleus in the same way, thereby reducing its expression and promoting cancer progression (73). As a well-known tumor suppressor, the expression of PTEN is significantly negatively regulated by LncRNA Kcnq1ot1 in triple-negative breast cancer. The specific mechanism is that the overexpression of lncRNA Kcnq1ot1 can enrich DNMT1 to increase the methylation ratio of PTEN promoter, thus inhibiting the expression of PTEN and promoting the progress of TNBC (74). ncRNAs usually recruit DNMTs to perform methylation in the promoter region of downstream targets, but Li et al. found that McL-1 can target the promoter of miR-142-5p and promote its transcription. The up-regulated miR-142-5p directly binds to the 3’-UTR of DNMT1, and then inhibits its expression, thereby indirectly promoting the expression of Maspin and inhibiting the progression of cancer (77). piR-651 increased DNMT1 enzyme activity, and further studies found that DNMT1 bound to the promoter region of the tumor suppressor gene PTEN, resulting in decreased expression and promoting cancer progression (78). miR-145 inhibits the expression of DNMT3A by directly binding to the 3’-UTR of its mRNA, and circIQCH downregulates miR-145 by function as its sponge, so as to relieve its negative regulation of DNMT3A and promote cancer progression (79).

miR-770-5p binds to 3’-UTR of DNMT3A mRNA and down-regulates DNMT3A, thereby reducing promoter methylation of CDH1 and increasing its expression, thus playing a role in cancer inhibition (80). lncRNA H19 can regulate the autophagy related gene Beclin1 to exert its carcinogenic effect of drug resistance. In particular, the Beclin1 promoter region was less methylated in tamoxifen-resistant cancer cells than in non-resistant cells. Mechanically, H19 can bind to and inhibit S-adenosine homocysteine hydrolase (SAHH), thereby reducing DNMT3B-mediated methylation, resulting in hypomethylation in the downstream Beclin1 promoter region, and up-regulating Beclin1 expression (81). lncRNA RAMP2-AS1 can recruit DNMT1 and DNMT3B to the CXCL11 promoter region and form a ternary complex, thereby promoting the methylation of CXCL11 and reducing its expression. Low expression of RAMP2-AS1, a cancer suppressor, impairs the inhibition of downstream CXCL11, leading to cancer progression (84). As a carcinogen, LINC01270 is highly expressed in breast cancer tissues and mainly concentrated in the nucleus of cancer cells. At the same time, LINC01270 binds to the promoter of the LAMA2 gene and recruits DNMT1, DNMT3A, and DNMT3B, resulting in high methylation of the LAMA2 promoter region, thus reducing its expression. Furthermore, cancer progression is promoted by weakening LAMA2 inhibition of MAPK signaling pathways (85). lncRNA MIAT recruits DNMT1, DNMT3A, and DNMT3B to the promoter region of the downstream target DLG3, and thus promotes the methylation of DLG3, leading to the down-regulation of its expression. Conversely, MIAT silencing can lead to up-regulation of DLG3, which in turn exerts its anticancer effect by activating the Hippo signaling pathway (86). LINC00922 binds to DNMT1, DNMT3A, and DNMT3B and is enriched in the promoter region of the downstream tumor suppressor NKD2, thereby inhibiting its expression through methylation, which ultimately activates the Wnt signaling pathway and promotes cancer progression (87). High expression of LINC00518 is accompanied by enrichment of CDX2 promoter region methyltransferase DNMT1, DNMT3A and DNMT3B, which negatively regulates its expression. Overexpressed CDX2 blocks the Wnt signaling pathway, and LINC00518 enhances activation of the Wnt pathway through methylation inhibition of CDX2 (90). LINC00472 plays a tumor suppressor role in triple-negative breast cancer. Overexpression of LinC00472 can recruit DNMT1, DNMT3A and DNMT3B to mediate the methylation of oncogenic factor MCM6, which leads to the down-regulation of its expression level, and then inhibits cancer progression by inactivating MEK/ERK signaling pathway (89). Activation of the nuclear factor erythroid 2 associated factor 2 (NRF2) led to down-regulation of miR‐29b‐1‐5p, increased cell proliferation and decreased intracellular ROS levels. Further studies showed that overexpression of miR‐29b‐1‐5p inhibited DNMTs (DNMT1, DNMT3A, and DNMT3B) and promoted the expression of some cancer suppressor factors (88). However, the specific mechanism of miR-29b-1-5p regulating DNMTs needs to be further revealed. xu et al. demonstrated that the tumor suppressor MAGI2−AS3 may regulate the expression of MAGI2 through a cis-acting mechanism, and overexpression of MAGI2‐AS3 leads to a reduction in the number of MAGI2 methylated CpG sites, leading to the up-regulation of MAGI2. The above anti-tumor effects have been shown to be achieved through partial inhibition of AKT signaling pathway, Wnt signaling pathway, and BAX/BCL2 apoptotic pathway (91).

#### Active regulation of ncRNAs by DNA methylation modification enzymes

The tumor suppressor LncRNA MEG3 is regulated by DNMT1’s hypermethylation in breast cancer, resulting in down-regulation of its expression. When the demethylation reagent 5-AzadC is applied, the hypermethylation regulation of MEG3 by DNMT1 is significantly inhibited, and uninhibited MEG3 exerts its inhibitory effect on cancer through the inhibition of Notch1 signaling pathway (75). UXT, a putative member of the alpha pre-folded protein family, has recently been found to be involved in the regulation of methylation. UXT is highly expressed in breast cancer and combines with DNMT3B to participate in the methylation regulation of MEG3, thus reducing the expression level of MEG3. At the same time, UXT knockdown can lead to the increase of MEG3 level, and MEG3 subsequently increases the expression of P53 to exert its anticancer effect (82, 83). lncRNA PHACTR2-AS1(PAS1), as the upstream regulatory gene of PH20, can inhibit its expression and reduce the aggressiveness of tumor cells. However, the expression of PAS1 is regulated by DNMT1 and leads to the decrease of the expression level, which undermines the inhibition effect of PAS1 on PH20 and leads to the progression of cancer (76).

In addition to single DNA methylation modification, the expression of ncRNAs is also regulated through the combination of multiple epigenetic modifications. LncRNA MIR497HG is regulated by various factors in breast cancer, in which ZEB1 and ERα play important roles. When breast cancer cells are in the state of endocrine resistance, ZEB1 recruits DNMT3B and HDAC1/2 to the MIR497HG promoter region, which leads to transcriptional repression through DNA methylation and histone modification. In addition, ERα also binds to the promoter region of MIR497HG in the endocrine sensitive state and promotes its expression through trans activation. Then miR-195 and miR-497 derived from MIR497HG reverse regulate the downstream PI3K/AKT pathway, playing a double-sided role in breast cancer (92).

### Crosstalk of ncRNAs and RNA methylation in BC

More than 150 RNA modifications have been identified, and the most studied are m6A, m5C, m1A, m7G and other methylation modifications. Among them, m6A is the most abundant methylation modification form in mRNA, and it is also the most well studied type of RNA modification (130, 131). In breast cancer, the crosstalk between ncRNAs and RNA methylation modification is also mainly concentrated in m6A. In this subsection, we provide a brief introduction to the crosstalk between ncRNAs and m6A modifications.

#### Active regulation of RNA methylation by ncRNAs

Ruan et al. found that METTL3 and m6A levels showed low expression and played a potent oncogenic role, which was mainly negatively regulated by the upstream target miR-34c-3p. In addition, the low expression level of circMETTL3, a sponge of miR-34c-3p, also indirectly promoted the expression of miR-34c-3p (93). ZNF217 could bind to METTL3 and prevent its m6A methylation on NANOG, thereby promoting its expression. miR-135 is a tumor suppressor, which can target and negatively regulate ZNF217, thereby promoting the m6A modification of NANOG and reducing its expression (100). By recognizing the specific recognition sequence, LNC942 directly binds to METTL14 and plays a positive regulatory role on METTL14 at the mRNA and protein levels, eventually affecting the methylation level of m6A in BRCA cells, and enhancing the mRNA stability and increasing the expression of its downstream targets CXCR4 and CYP1B1 through m6A modification (101). lncRNA UCA1 recruits DNMT1, DNMT3A, and DNMT3B in the promoter region of METTL14 to promote its DNA methylation and reduce its expression. METTL14 can reduce the expression of miR-375 through m6A modification and indirectly promote the expression of downstream SOX12, thereby playing a tumor suppressor role. Mechanistically, UCA1 reduced m6A modification in breast cancer cells in a DNA methylation modification manner (102). piRNA-17560 binds to the 3’-UTR of FTO to reduce the decay and enhance the stability of FTO mRNA, and then FTO up-regulates the expression of ZEB1 through m6A demethylation. Moreover, YTHDF2 directly binds to the mRNA of ZEB1 and enhances its stability and expression. Ultimately, this dual dem6A methylation collectively promotes chemoresistance in BC cells (105). m6A methyltransferase WTAP increases the expression of DLGAP1-AS1 by promoting its stability, and indirectly upregulating WTAP by targeting miR-299-3p, thereby forming a positive feedback loop and increasing the resistance of BC cells to ADR (107).

#### Active regulation of ncRNA by RNA methylation modification enzymes

As an m6A modification enzyme, KIAA1429 enhances mRNA stability and promotes its expression by targeting the m6A modification site of LINC00667. In addition, LINC00667 positively regulates KIAA1429 through sponging miR-556-5p, thus forming a positive feedback loop and promoting cancer progression (132). Rong et al. confirmed the existence of m6A modification site on LINC00958 by Merip-Seq analysis and demonstrated that METTL3 was involved in the enrichment of m6A modification, resulting in the overexpression of LINC00958 through knockdown and overexpression experiments. Indirectly inhibited the downstream target miR-378a-3p and increased YY1 expression to promote cancer progression (94). Guo et al. used the SRAMP tool to find that the m6A methylation modification site of LINC00520 is GGACU, and m6A is highly enriched in tissues overexpressed by LINC00520. In addition, the expression level of METTL3 is closely related to LINC00520, and the m6A modification of LINC00520 can be actively regulated and its expression can be increased. Subsequently, overexpressed LINC00520 targets miR-577, leading to up-regulation of downstream target gene POSTN, thereby promoting the ILK/Akt/mTOR pathway (95). As a tumor suppressor, the m6A site of LINC00675 is located in AGACA, and METTL3 knockdown can significantly decrease the m6A level of LINC00675 and lead to down-regulation of its expression, which indirectly leads to the up-regulation of oncogenic factor miR-513b-5p (96). Lei et al. found that LINC00662 was significantly enriched in m6A, and further studies confirmed that METTL3 could bind to LINC00662 and positively regulate its expression, thus inhibiting the downstream target miR-186-5p. Interestingly, overexpressed miR-186-5p inhibited the expression of METTL3 in turn. Therefore, Lei et al. speculated that LINC00662 improves the expression of METTL3 through sponging miR-186-5p to form a positive feedback loop that promotes the expression of LINC00662 (97). METTL3 promotes the expression of MALAT by increasing its m6A modification level. Mechanically, it up-regulates MALAT1 sponging and inhibits the expression of miR-26b, thus promoting HMGA2 and promoting cancer progression (98). circMETTL3 was found to be highly enriched in m6A modification, and knockdown of METTL3 could reduce the expression of circMETTL3 and the level of m6A modification, and indirectly weaken its carcinogenic effect through the circMETTL3/miR-31-5p/CDK1 axis (133). METTL3 could enhance the m6A methylation of pri-miR-221-3p, thereby increasing the expression of miR-221-3p and increase the drug resistance of breast cancer cells through the miR-221-3p/HIPK2/Che-1 axis (134). METTL14 increases the expression of has-miR-146a-5p by regulating m6A modification, thereby promoting the migration and invasion of cancer cells (103). FTO, as an m6A demethylase, is highly expressed only in HER2-positive cell lines and can significantly down-regulate the level of m6A. The most affected is miR-181b-3p, whose expression is significantly inhibited, thereby indirectly upregulating downstream ARL5B and promoting cancer (104). LncRNA FGF14 is a key repressor of breast cancer metastasis. FGF14-AS2, as an antisense lncRNA transcribed from the opposite strand of FGF14, can significantly inhibit the osteolytic metastasis of breast cancer by down-regulating the RUNX2/RANKL axis. Furthermore, the overexpression of YTHDF2 promoted the rapid degradation of m6A-modified FGF14-AS2, thereby exerting a tumor suppressor effect (106).

### Crosstalk of ncRNAs and Histone modification in BC

Histone proteins and DNA constitute the basic structure of chromatin. Histone modifications (such as acetylation, methylation, and phosphorylation) regulate chromatin structure and gene expression by regulating chromatin compaction (129). In this subsection, we will explore the mutual regulatory mechanism between ncRNAs and histone modifications.

#### ncRNAs actively regulate histone methylation

As a histone lysine methyltransferase, SUV420H2 can specifically trimethylate Lys-20 of histone H4, resulting in transcriptional repression of related genes. miR-29a can directly target SUV420H2 and inhibit its expression, leading to the inhibition of trimethylation of H4K20 and ultimately promoting cancer progression (108). SNHG1 can bind to EZH2, the subunit of PRC2, and target miR-381 at the same time. EZH2 then binds to the promoter region of miR-381, resulting in H3K27me3 modification, thereby down-regulating its expression and weakening its anti-tumor effect (109). lncRNA UCA1 can recruit EZH2 to the promoter region of P21, increase H3K27me3, and thus reduce its expression (112). Cullin 4B (CUL4B) can simultaneously recruit CRL4B, PRC2 and HDAC to the promoter region of miR-32-5p and increase H2AK119ub1 and H3K27me3 to inhibit its transcription (110). SETD1A is a histone H3 lysine 4-specific methyltransferase, which can maintain the histone H3K4 methylation of ERα target gene trefoil factor 1 (TFF1) and increase the occupancy of ERα in TFF1 enhancer and promoter regions in ER-positive breast cancer cells. On the contrary, depletion of SETD1A resulted in a significant reduction in the recruitment of ERα. Further studies demonstrated that miR-1915-3p directly bound to the 3’-UTR of SETD3A mRNA and subsequently reduced SETD1A protein level, exerting a tumor suppressor effect (111). Exosomal miR-138-5p, which is upregulated in breast cancer, prevents the expression of histone demethylase KDM6B, which reduces the level of H3K27me3, thereby increasing the level of H3K27me3 and the transcriptional activity of genes encoding proinflammatory factors, leading to the suppression of M1 polarization (113). LncRNA ROR can recruit methyltransferase MLL1 to the promoter region of TIMP3, which then enriches H3K4me3, thereby increasing TIMP3 transcription level (114).

#### ncRNAs actively regulate acetylation in promoter regions

Histone deacetylase 3 (HDAC3) is a potential oncogene. Rahbari et al. found that it is up-regulated in TNBC and negatively regulated by its upstream target miR-589-5p. However, the specific downstream target genes regulated by HDAC3 have not been further determined (116). xie et al. found 8 lncRNAs with high H3K27 acetylation peaks in the non-coding genome, among which RP11-162G10.5 had the highest expression, which was closely related to the poor prognosis of patients (127). Zhou et al. demonstrated that lncRNA HULC can increase H3K9 acetylation in the promoter region of IGF1R in the nucleus, thereby activating IGF1R transcription, which in turn causes activation of the downstream PI3K/AKT pathway (121). DLEU1 is highly expressed in breast cancer and is subject to two epigenetic modifications to regulate its expression. Besides the hypomethylation of DLEU1 promoter, H3K27ac and H3K4me3 were also enriched at DLEU1 locus. Upregulated DLEU1 then promotes downstream SRP4 expression by increasing H3K27ac enrichment (122). Overexpression of LINC00473 enriched H3K27ac at the CRE region, and further study confirmed that LINC00473 activated cyclin D1 (CCND1) expression by recruiting phosphorylated CREB and histone acetylation to the CCND1 promoter site (120). HDAC2 acts as a transcriptional corepressor by inducing deacetylation of the PLIN1 promoter; conversely, depletion of HDAC2 renders the PLIN1 gene promoter highly acetylated, ultimately enhancing PLIN1 transcription in BC cells. As a ceRNA, ARAP1-AS1 increased the expression of HDAC2 by competitive binding to miR-2110, and thus inhibited the expression of PLIN1 (115). HER2/neu signaling inhibits p65Ser536 phosphorylation, which results in transcriptional activation of miR-22. miR-22 further negatively regulates the expression of transcriptional repressors FOXP1 and HDAC4 and causes changes in histone and p53 acetylation (119). lncRNA SNHG14 induces PABPC1 up-regulation by regulating H3K27 acetylation in the PABPC1 promoter region (123). The oncogenic factor LINC01857 promotes H3K27Ac and CREB1 transcription by promoting the enrichment of CREBBP in the CREB1 promoter region, thereby promoting cancer progression (124). STAT3 positively activated CASC9 transcription, and CASC9 subsequently upregulated SOX4 expression by inducing H3K27 acetylation and up-regulating its promoter region acetylation (126). miR-590-3p can directly increase the acetylation of p53 promoter region and increase its expression, thereby up-regulating the expression of downstream tumor suppressor factors BAX and p21 (125).

#### ncRNAs actively regulate histone phosphorylation

miR-941 was found to be highly expressed and play a carcinogenic role in breast cancer. Further studies confirmed that inhibition of miR-941 could reduce the expression of histone H3 ser10 phosphorylation, thereby inhibiting the proliferation of cancer cells (128).

## Biological effects of ncRNAs involved in epigenetic modification on BC

**Figure 3:**
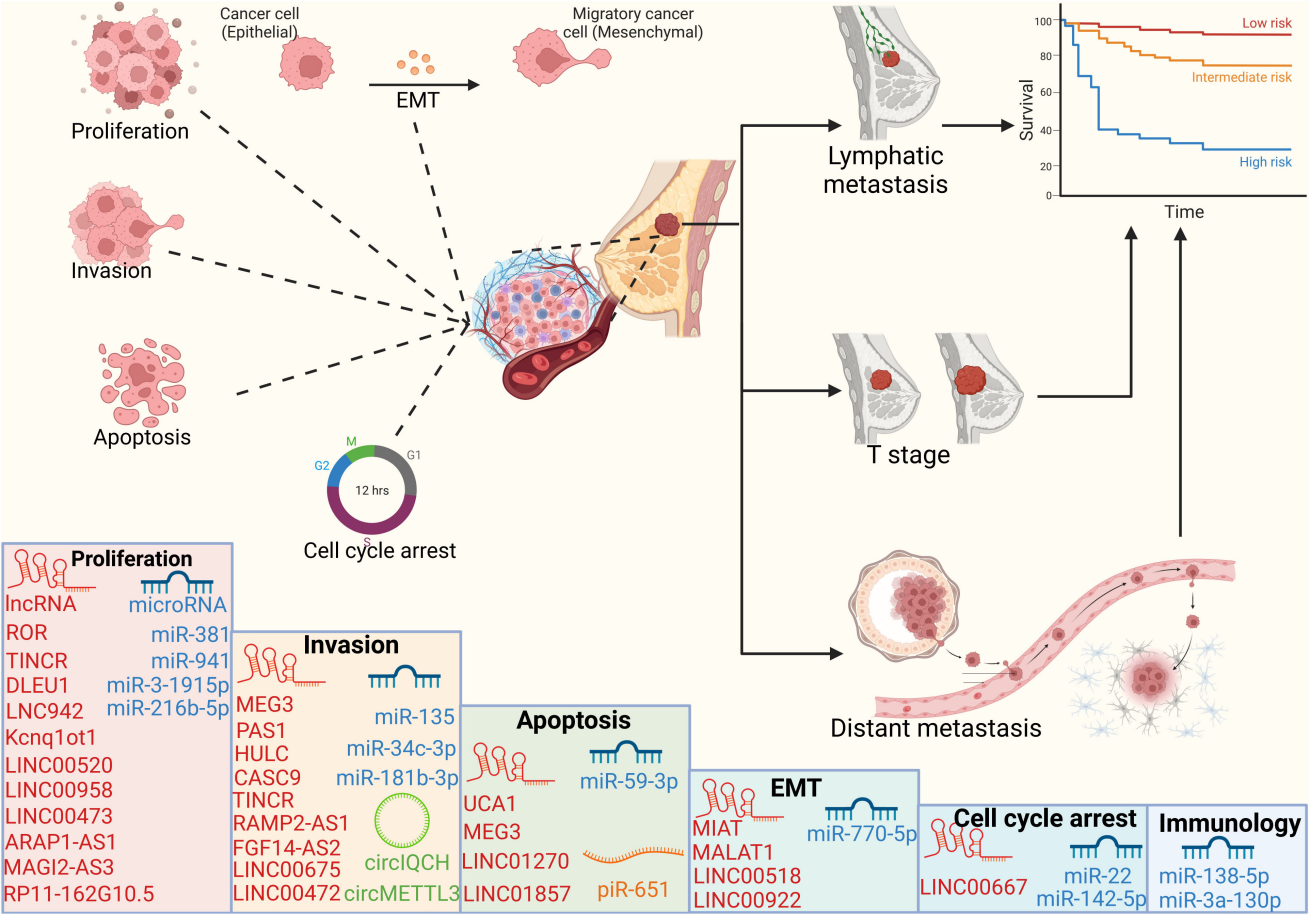
Cellular processes and prognosis regulated by ncRNAs ncRNAs can regulate cell proliferation, apoptosis, invasion and cell cycle, thereby affecting the tumor volume, lymph node metastasis and distant organ metastasis of breast cancer patients, and ultimately affecting the survival cycle of patients.

The bulk of the human transcribed genome is made up of non-coding RNAs (ncRNAs), which also include microRNAs, lncRNAs, circRNAs, and piRNAs. These ncRNAs play various functions in many cellular processes and are linked to a variety of adverse outcomes in cancer (135). We give a thorough summary of the ncRNAs that have an impact on biological processes in breast cancer through epigenetic alterations in this subsection (**Figure 3**).

### LncRNA in BC

Recent studies have confirmed the important role of lncRNA in the occurrence and development of breast cancer, especially the regulation of its biological function by abnormal expression of lncRNA (136–138). In this subsection, we will further elaborate on the functional mechanisms involved in lncRNA, including cell proliferation, invasion and metastasis, cell cycle and EMT (**Table 2**).

**Table 2:** The effect of lncRNA on the biological behaviors of breast cancer and its correlation with prognosis.

lncRNA	Role in breast cancer	*In vitro*	*In vivo*	Clinical prognosis	Ref.
TINCR	carcinogen	Promote proliferation, colony formation and invasiveness	Tumor volume and weight	TNM stages III and IV, poor overall survival	(72)
TINCR	carcinogen	Promote proliferation, invasiveness and migration	Tumor weight	/	(73)
lncRNA Kcnq1ot1	carcinogen	Promote cell viability, migration, and invasion	Tumor volume and weight	Poor overall survival	(74)
LINC01270	carcinogen	Promote proliferation, migration and invasion, decrease apoptosis rate	Tumor growth	Poor overall survival	(85)
LncRNA MEG3	tumor suppressor	Inhibit EMT, proliferation and induces apoptosis	Tumor growth	/	(75)
lncRNA MIAT	carcinogen	Promote cell viability, migration and invasion, and EMT	Tumor formation	3-year survival rate, stage III	(86)
MAGI2‐AS3	tumor suppressor	Inhibit cell proliferation, migration, and promotes apoptosis	/	Longer survival time	(91)
lncRNA H19	carcinogen	Promote autophagy	Tumor volume and weight	/	(81)
MEG3	tumor suppressor	Inhibit proliferation, promote cell apoptosis, delay migration and invasion	Tumor growth	Tumor size, Lymph node metastasis and TNM stage	(82)
RAMP2-AS1	tumor suppressor	Inhibit viability, proliferation, migration and invasion, and promote cell apoptosis	Tumor growth	Poorly differentiated and Lymphatic metastasis	(84)
LINC00922	carcinogen	Promote cell viability, invasion and metastasis. Promote EMT	Enhance breast cancer metastasis to lung and liver	Tumor size and TNM stage	(87)
LINC00518	carcinogen	Promote proliferation, invasion and migration. Promote EMT. Inhibit epithelial cell apoptosis	Promote tumor growth and metastasis	/	(90)
LncRNA PHACTR2-AS1	tumor suppressor	Decreased the migration ability	Inhibit tumor growth and metastasis	/	(76)
LINC00472	tumor suppressor	Inhibit cell proliferation, migration and invasion	Tumor volume and weight, Pulmonary metastatic nodules	Poor differentiation, III Clinical grade, Lymph node metastasis	(89)
LINC00667	carcinogen	promote the proliferation, migration, and accelerate the cycle progression	Promote tumor growth	Poor overall survival	(132)
LINC00958	carcinogen	Promote proliferation and inhibit apoptosis	Promote tumor growth	Advanced stage (III–IV grade) and Poor overall survival	(94)
LINC00520	carcinogen	promote the proliferation and migration	Promote tumor growth	Advanced stage (III–IV grade), poor differentiation and poor overall survival	(95)
LINC00675	tumor suppressor	Inhibit BC cell proliferation, migration and invasion	/	tumor grade, overall survival and lymphovascular invasion	(96)
MALAT1	carcinogen	Promote EMT, invasion and migration	Increase tumorigenesis and metastasis	Poor overall survival	(98)
LNC942	carcinogen	Promote cell proliferation, colony formation, metastasis, and inhibit apoptosis	Promote tumor growth	/	(101)
LncRNA UCA1	carcinogen	Promote the proliferation and invasion, inhibit apoptosis	Promote tumor growth	shorter survival time	(102)
FGF14-AS2	tumor suppressor	/	Inhibit osteolytic metastasis	distant metastasis-free survival	(106)
ROR	carcinogen	Promote cell proliferation and invasion, and inhibit apoptosis	Promote tumor growth	/	(114)
lnc RP11-162G10.5	carcinogen	Promote cell proliferation	Promote tumor growth	tumor size and TNM stage	(127)
lncRNA HULC	carcinogen	promote the proliferation and migration	Enhance breast cancer metastasis to lung	lymph node metastasis and distant metastasis	(121)
DLEU1	carcinogen	Increase cell viability and clone formation	Tumor volume and weight	/	(122)
LINC00473	carcinogen	Promote the proliferation	/	/	(120)
ARAP1-AS1	carcinogen	Promote the proliferation and migration	/	/	(115)
LINC01857	carcinogen	Promote proliferation and inhibit apoptosis	/	lymph node metastasis, large tumor size, and advanced clinical stage	(124)
LncRNA CASC9	carcinogen	Promote the proliferation and migration	/	tumor volume, TNM stage and lymph node metastasis	(126)

#### lncRNA regulates proliferation

Up-regulation of LNC942 could significantly promote BRCA cell proliferation, colony formation, metastasis, as well as inhibit apoptosis through METTL14/CXCR4/CYP1B1 signaling pathway axis, and significantly promote tumor growth in mice (101).

Low expression of TINCR significantly inhibited tumor volume and weight *in vivo*, and had the same inhibitory effect on cell proliferation, as well as reducing the aggressiveness of tumor cells. In terms of mechanism, TINCR can act as a ceRNA to sponge miR-503-5p, thereby up-regulating downstream EGFR expression and downstream signal JAK2 and STAT3 conduction, ultimately leading to a malignant phenotype of breast cancer (72). Shen et al. initially found that yin yang1 (YY1), a protein in the zinc finger transcription factor family, was highly expressed in triple-negative breast cancer tissues, and its expression level was inversely proportional to the overall survival of patients. Further studies found that YY1 could weaken the expression of downstream cancer suppressor PTEN by up-regulating the expression level of lncRNA Kcnq1ot1, thus promoting the adverse progression of TNBC. *In vitro* experiments demonstrated that both upregulated YY1 and lncRNA Kcnq1ot1 could improve the viability of TNBC cells (74). Up-regulated LINC00958 promotes tumor cell proliferation and promotes tumor growth in mice through the LINC00958/miR-378a-3p/YY1 axis. In addition, high expression of LINC00958 in patients is strongly associated with Advanced stage (III – IV grade) and poor overall survival (94). MAGI2‐AS3, a lncRNA transcribed from an antisense chain near the MAGI2 gene, acted as a tumor suppressor gene in breast cancer. Overexpression of MAGI2-AS3 was confirmed to prevent cell proliferation, metastasis and promote apoptosis of cancer cells, and was in direct proportion to longer survival of patients (91). Overexpression of LINC00520 inhibits the expression of downstream tumor suppressor miR-577, up-regulate POSTN, and promote the activation of ILK/AKT/mTOR pathway. Knockdown of LINC00520 can significantly inhibit cell proliferation and tumor growth. In addition, overexpressed LINC00520 was strongly associated with Advanced stage (III – IV grade), poor differentiation, and poor overall survival (95). lncRNA ROR can enhance cell proliferation and invasion ability by increasing the transcription expression of TIMP1. Silencing TIMP1 can reverse the carcinogenic effect of ROR. Similarly, ROR depletion effectively suppressed breast cancer tumor growth *in vivo* (114). The high expression of RP11-162G10.5 was closely related to tumor size and TNM stage. Mechanistically, RP11-162G10.5 recruited transcription factor YBX1 to the GLO1 promoter and activated the transcription and expression of GLO1, thereby promoting the proliferation *in vitro* and *in vivo* (127). DLEU1 can promote cell viability and colony formation, and high expression of DLEU1 is associated with larger tumor size and weight *in vivo* (122). LINC00473 was upregulated in breast cancer in a camp-dependent manner and promoted cell proliferation by regulating CCND1 transcription (120). ARAP1-AS1 inhibits the expression of PLIN1 at the transcriptional level and promotes the proliferation and migration of BC cells (115).

#### lncRNA regulates invasion and metastasis

Wang et al. found that STAT1 was significantly enriched in the promoter region of TINCR and directly bound to it, thereby upregulating TINCR expression. Then, TINCR recruits USP20 for de-ubiquitination of PD-L1 and promotes its expression at the protein level, thus increasing the invasion and metastasis ability (73). MEG3 is down-regulated in breast cancer and is closely related to tumor size, lymph node metastasis, and TNM stage. *In vitro* experiments showed that its overexpression could impair migration and invasion. *In vivo* experiments have demonstrated that overexpressed MEG3 can effectively inhibit tumor growth (82). The low expression of RAMP2-AS1 as a tumor suppressor in breast cancer is closely related to poor differentiation and lymph node metastasis. Experiments showed that its overexpression could effectively inhibit the viability, proliferation, invasion and metastasis of breast cancer cells, and promote apoptosis. It can effectively inhibit tumor growth *in vivo*. This is achieved by inhibition of the downstream oncogene CXCL11 by RAMP2-AS1 (84). PH20, a member of the human hyaluronidase family, promotes tumor cell metastasis by degrading hyaluronic acid in the extracellular matrix. PAS1 reduced the tumor volume by inhibiting the expression of PH20, and effectively reduced the number of lung metastatic nodules. *In vitro* experiments also confirmed that PAS1 knockdown enhanced the migration ability, but after knocking down PH20, the cell migration ability was inhibited again (76). LINC00472 is down-expressed in patients with triple-negative breast cancer and is significantly associated with poor pathological differentiation, lymph node metastasis, and clinical grade. *In vitro* studies demonstrated the inhibitory effect of overexpressed LINC00472 on cell invasion and metastasis, mainly through inhibition of MCM6 and MEK/ERK pathways. Meanwhile, *in vivo* experiments also confirmed that LINC00472 effectively inhibited lung nodule metastasis and tumor volume and size in mice by down-regulating MCM6 (89). Low expression of LINC00675 is associated with higher tumor grade and lymphovascular infiltration, and shorter patient survival. *In vivo* experiments have demonstrated that overexpression of LINC00675 can effectively inhibit cell proliferation, invasion and metastasis (96). FGF14-AS2 significantly inhibited osteolytic metastasis by down-regulating the RUNX2/RANKL axis. Mechanistically, FGF14AS2 inhibits RUNX2 translation by inhibiting eIF4E/eIF4G complex assembly and eIF4E phosphorylation, thereby reducing transcription of RANKL, a key regulator of osteoclast differentiation. Low expression of FGF14-AS2 is closely related to distant metastasis-free survival of patients (106). The nuclear lncRNA HULC plays a role in promoting cancer metastasis *in vivo* and *in vitro* by activating the IGF1R/PI3K/AKT pathway. The overexpression of HULC could significantly enhance the invasion and metastasis of breast cancer cells, and the same idea was also verified in the mouse lung metastasis model. In addition, HULC has also been shown to be positively correlated with lymph node metastasis and distant metastasis in patients (121). LncRNA CASC9 is highly expressed in BC tissues and cell lines, and is positively correlated with tumor volume, TNM stage and lymph node metastasis. Moreover, CASC9 silencing significantly inhibited cell invasion, as well as BC-associated human dermal lymphatic endothelial cell invasion and lymphangiogenesis. Mechanistically, CASC9 enhanced the oncogenic ability of SOX4 mainly by increasing its expression (126).

#### lncRNA regulates apoptosis

LINC01270 inhibits the expression of LAMA2 to enhance the proliferation, invasion and metastasis of tumor cells, and reduces their apoptosis rate. Mechanically, the outcome of this malignant behavior is caused by the impaired ability of LAMA2 to inhibit MAPK pathway activation. Therefore, LAMA2 can inhibit the growth of tumor grafts by inhibiting the activation of MAPK pathway after silencing the expression of LINC01270 (85). The expression of LncRNA MEG3 is lower in breast cancer. With the knockdown of the upstream inhibitory target DNMT1, the highly expressed MEG3 shows high potential for cancer inhibition, including inhibiting cancer cell proliferation and promoting apoptosis, and inhibiting EMT through Notch1 signaling pathway. MEG3 can also effectively inhibit tumor growth in nude mice tumor grafts (75). Patients with up-regulation of LncRNA UCA1 often have a shorter survival time. *In vitro* experiments, the oncogenic effect of UCA1 is mainly reflected in promoting cell proliferation, enhancing invasion and inhibiting apoptosis (102). Xiong et al. found that high expression of LINC01857 was positively correlated with lymph node metastasis, tumor size, and advanced clinical stage. *In vitro* experiments demonstrated that LINC01857 knockout could effectively promote cell apoptosis, mechanically, this is achieved through the activation of the downstream CREB1 (124).

#### LncRNA regulates EMT

The 3-year survival rate of breast cancer patients was inversely correlated with the level of MIAT expression, and stage III patients expressed higher MIAT and lower DLG3 than Stage I-II. Upregulated MIAT exerts its carcinogenic effect by inhibiting downstream cancer suppressor DLG3, whereas silencing MIAT inhibits cell viability, migration and invasion, and EMT. At the same time, silencing MIAT significantly inhibited the xenograft volume and weight in nude mice. Mechanistically, upregulated DLG3 inhibits cancer progression by activating downstream Hippo signaling pathways (86). The high expression of LINC00922 is closely related to the TNM stage and tumor size of patients. LINC00922 enhances cell viability and promotes the invasion and metastasis of cancer cells by inhibiting the downstream target NKD2, and also promotes EMT by activating the Wnt signaling pathway. *In vivo* studies have also demonstrated that LINC00922 promotes tumor graft growth and enhances lung and liver metastasis of breast cancer by negatively regulating NKD2 (87). LINC00518 is associated with poor prognosis of patients, and its low expression can promote the apoptosis of breast cancer epithelial cells and inhibit the proliferation, invasion and metastasis of cancer cells by increasing the expression of CDX2. In addition, highly expressed CDX2 can inhibit EMT by inactivating the Wnt signaling pathway. All of these antitumor effects were reversed by up-regulated LINC00518. *In vivo* studies have demonstrated that LINC00518 can promote tumor growth and distant metastasis (90). MALAT1 is highly expressed in breast cancer and negatively correlated with the survival of patients. Knockdown of MALAT1 inhibited EMT, invasion and migration of BC cells *in vitro*. Similarly, *in vivo* experiments demonstrated the potent pro-metastatic ability of MALAT1 by up-regulating HMGA2 (98).

#### lncRNA regulates cell cycle

KIAA1429 enhanced the mRNA stability of LINC00667 and promoted its expression. High expression of LINC00667 can promote cancer cell proliferation, migration and accelerate cell cycle. On the contrary, knockdown of LINC00667 can significantly inhibit the above malignant behavior and block cancer cells in G1/S phase. *In vivo* experiments also confirmed that LINC00667 can promote tumor growth in mice (132).

### miRNAs in BC

As a kind of small ncRNA, miRNA can play an important role in regulating the expression of downstream target genes, and most of them are competitively regulated by other lncRNAs (139–141). The aberrant expression of miRNAs has been widely confirmed to play a crucial role in different biological behaviors in breast cancer (**Table 3**).

**Table 3:** The effects of miRNAs, piRNAs and circRNAs on the biological behaviors of breast cancer.

ncRNA	Role in breast cancer	*In vitro*	*In vivo*	Ref.
miR-142-5p	tumor suppressor	Inhibit cell migration and proliferation. G1 phase arrest	Tumor volume and growth	(77)
miR-770-5p	tumor suppressor	inhibit the mobility and invasion ability, and significantly inhibit EMT	/	(80)
miR-34c-3p	carcinogen	promotes the proliferation, migration and invasion	Promote tumor growth and metastasis	(93)
miR-135	tumor suppressor	Inhibit EMT initiation, migration and invasion	Suppress tumor growth and metastasis	(100)
miR-181b-3p	tumor suppressor	Inhibit migration and invasion	/	(104)
miR-29a	carcinogen	Promote the migration and invasion, promote EMT	Promote tumor growth and metastasis	(108)
miR-381	tumor suppressor	Inhibit the proliferation and invasion, inhibit apoptosis	/	(109)
miR-1915-3p	tumor suppressor	Inhibit cell proliferation and migration	Reduce the growth rate	(111)
miR-138-5p	carcinogen	Promotes the polarization of M2 macrophages	Promote lung metastasis	(113)
miR-589-5p	tumor suppressor	inhibits cell proliferation, colony formation, and cell cycle	/	(116)
miR-216b-5p	tumor suppressor	inhibits cell proliferation	/	(118)
miR-22	tumor suppressor	induces S phase arrest and apoptosis	/	(119)
miR-130a-3p	tumor suppressor	restrains cell viability, migration, EMT and facilitates apoptosis, and anti-CD8+/CD69+/PD-1+T cytotoxicity	/	(117)
miR-590-3P	tumor suppressor	inhibited the cell viability and promoted apoptosis	/	(125)
miR-941	carcinogen	promotes the proliferation, migration and invasion	/	(128)
circIQCH	carcinogen	Promote cell proliferation and metastasis	Promote lung metastasis	(79)
piR-651	carcinogen	Promote cell proliferation and invasion, inhibit cell apoptosis	/	(78)
circMETTL3	carcinogen	Promote proliferation and enhance migration and metastasis	/	(99)

#### miRNA regulates proliferation

As a tumor suppressor, miR-381 is negatively regulated by its upstream SNHG1, and low expression of miR-381 is often associated with shorter survival time of patients. *In vivo* experiments confirmed that overexpression of miR-381 inhibited tumor cell proliferation, invasion and metastasis, and improved DDP resistance (109). miR-3-1915p was down-regulated in ERα positive breast cancer cells, while SETD1A, which was negatively regulated by miR-3-1915p, was up-regulated. *In vitro* experiments showed that SETD1A depletion significantly inhibited cell proliferation and migration. In addition, SETD1A knockdown also significantly reduced the growth rate of xenografts in mice (111). The expression of miR-216b-5p is low in breast cancer tissues and negatively correlated with lymph node metastasis and tumor size. miR-216b-5p can directly bind to the 3’-UTR of HDAC8 mRNA and significantly inhibit its expression level, which effectively inhibits the malignant proliferation of breast cancer cells (118). As a carcinogenic factor, inhibition of miR-941 expression can significantly reduce the expression level of cyclin D1 and increase the expression level of P21 and inhibit the proliferation of BC cells. In addition, inhibition of miR-941 could also increase the expression of E-cadherin and reduce the expression of MMP-13 protein, thereby inhibiting invasion and metastasis (128).

#### miRNA regulates invasion and metastasis

miR-34c-3p, an upstream regulator of METTL3, is able to bind directly to the 3’-UTR of its mRNA and down-regulate its expression, which then promotes the ability of migration and invasion. *In vivo* tumorigenic assays in nude mice also demonstrated that inhibition of miR-34c-3p expression significantly inhibited tumor growth and metastasis *in vivo* (93). As a tumor suppressor, miR-135 is poorly expressed in BC and closely related to lymph node metastasis. xu et al. found that overexpression of miR-135 could effectively inhibit EMT, invasion and migration abilities. In addition, miR-135 effectively suppressed tumor growth and metastasis *in vivo* by down-regulating ZNF217/NANOG (100). As a tumor suppressor, the overexpression of miR-181b-3p can significantly inhibit cell migration and invasion. However, miR-181b-3p is down-regulated in HER2-positive breast cancer cells due to FTO regulation (104).

#### miRNA regulates apoptosis

It has been established that miR-59-3p directly targets SIRT1, and that miR-59-3p dramatically reduces SIRT1 expression. In addition, miR-59-3p could up-regulate p53 and eventually lead to the up-regulation of BAX and p21, which significantly inhibited the cell viability and promoted apoptosis of BC cells (125).

#### miRNA regulates EMT

Overexpressed miR-770-5p can up-regulate the expression of CDH1, effectively inhibit the mobility and invasion ability of TNBC cells, and significantly inhibit EMT (80). As an oncogenic factor, miR-29a promotes EMT, invasion and metastasis of breast cancer cells by up-regulating EGR1 and CTGF. Moreover, miR-29a promoted lung metastasis of breast cancer cells *in vivo* by inhibiting SUV420H2 expression (108).

#### miRNA regulates cell cycle

miR-142-5p is positively regulated by the upstream target McL-1 and promotes the expression of maspin to play a tumor suppressor role and cause G1 phase arrest. *In vivo* experiments have confirmed that the tumor of nude mice in the group with high expression of miR-142-5p has a smaller volume and slower growth rate (77). Wang et al. found that miR-22 plays a dual role in fulvestrant-resistant breast cancer cells. Either inhibition or overexpression of miR-22 enhanced the expression of p21Cip1/Waf1 and p27Kip1, and thereby inhibited cell proliferation, induced apoptosis, and caused cell cycle S-phase arrest (119).

#### miRNA regulates immune cell

Macrophages are divided into a classically activated M1 phenotype and an alternatively activated M2 phenotype based on their differentiated surface receptor expression and function. M1 macrophages usually play an anti-tumor role, while M2-polarized macrophages are usually considered as tumor-associated macrophages (TAMs), which promote cancer progression through angiogenesis, lymphangiogenesis regulation and immunosuppression. miR-138-5p prevented M1 polarization and promoted M2 differentiation of macrophages by inhibiting the expression of KDM6B in macrophages, and significantly promoted lung metastasis of breast cancer in mice (113). HDAC3, as an oncogenic factor, can inhibit cell viability, migration, EMT and promote cell apoptosis by up-regulating the expression of miR-3a-130p. Furthermore, it is also found that HDAC3 could inhibit the proliferation and promote apoptosis of CD8+/CD69+/PD-1+T cells (117).

### circRNAs and piRNAs in BC

circIQCH is upregulated in metastatic breast cancer and has been shown to promote cell proliferation and metastasis *in vitro*. In addition, inhibition of circIQCH in lung metastasis in nude mice has been shown to reduce the number of lung metastatic nodules. The above carcinogenic effects were mainly achieved by circIQCH acting as a sponge for miR-145 (79). Li et al. found that circMETTL3 was stably and highly expressed in breast cancer tissues and could promote cell proliferation and enhance cell migration and invasion *in vitro* and *in vivo* (133). piR-651 is upregulated in breast cancer and plays a pro-cancer role, such as promoting cell proliferation and invasion, promoting cell cycle progression and inhibiting cell apoptosis by blocking G2/M phase, while reducing piR-651 has a significant anti-cancer effect (78).

#### Potential clinical application of ncRNAs in BC drug resistance

One of the most prevalent malignancies in women and the main cause of death is breast cancer. Despite significant attempts to enhance breast cancer early diagnosis and treatment effectiveness, the frequent emergence of drug resistance remains a prominent factor in cancer patients’ poor prognoses (142, 143). ncRNAs interact with a range of RNAs and proteins and are dysregulated in a number of cancers, ultimately leading to drug resistance (144, 145). As a result, ncRNAs associated with cancer resistance can be investigated as pertinent therapeutic targets and may offer new choices for patients with cancer resistance.

Programmed cell death -1 receptor (PD-1) is an important immunosuppressive molecule, which is activated by binding with PD-L1 and expressed on the surface of immune effector cells to inhibit the activation and proliferation of T cells, deplete the function of effector T cells, and enable tumor cells to achieve immune escape (146). It has been reported that lncRNA TINCR inhibits ubiquitination of PD-L1 through a dual mechanism of up-regulation of USP20 to ensure its stable expression at the protein level. In the cytoplasm, TINCR sponges miR-199a-5p to increase USP20 mRNA stability. In the nucleus, TINCR can attract DNMT1 to support the methylation of miR-199a-5p, which inhibits its transcription and lessens its inhibition of USP20 mRNA stability in the cytoplasm. This, in turn, causes the expression of PD-L1 to be upregulated, which induces immune escape (73). This mechanism fully demonstrates the potential of TINCR as an immunotherapeutic target for breast cancer.

Cytotoxic drugs are still the first line of treatment for metastatic cancer, but the emergence of evolving drug-resistant cancer cells is a major obstacle on the road to chemotherapy (147, 148). Autophagy is a physiological mechanism by which tumor cells can effectively avoid cell death and induce drug resistance (144, 149). Planned cell degradation and proper recovery of damaged organelles can effectively reduce cell stress caused by cytotoxic substances, thus avoiding cell death and generating resistant cancer cells (150). H19 is significantly upregulated in tissues and cell lines of tamoxifen-resistant patients and promotes autophagy of breast cancer cells by promoting Beclin1 expression. On the contrary, H19 knockdown can inhibit autophagy synthesis, reduce the formation of autophagosomes and autolysosomes, and induce G2/M cell cycle arrest. This was also confirmed in xenografts in nude mice, where inhibition of H19 expression inhibited autophagy and restored tamoxifen sensitivity, significantly inhibiting tumor volume and mass (81). The discovery of this target can provide a new therapeutic target for tamoxifen resistant patients in the future. MEG3, as a cancer suppressor, is regulated by methylation, resulting in decreased expression, especially in drug-resistant cell lines. The expression of MEG3 is restored by the combination of DNA methyltransferase inhibitor 5-aztidine, and the combination of MEG3 and chemotherapy agents in drug-resistant cell lines can significantly inhibit invasion and migration. This means MEG3 has the potential to be an important target for resistance (83). METTL3 increased the expression of miR-221-3p in ADR-resistant cells in a m6A mRNA methylation-dependent manner, and miR-221-3p in turn negatively regulated tumor suppressor HIPK2 to promote ADR-resistance. *In vivo* experiments confirmed that HIPK2 overexpression reduced the expression of Che-1, increased the expression of apoptotic protein Bax, decreased the expression of Bcl-2, and promoted cell apoptosis. In addition, overexpression of HIPK2 also reduced the expression of drug resistance proteins BCRP and MDR1, effectively inhibiting drug resistance. These tumor suppressive effects of HIPK2 could be inhibited by the overexpression of upstream miR-221-3p. Therefore, miR-221-3p is expected to be a new therapeutic target (134). METTL3 and LINC00662 promote each other’s expression in the form of a positive feedback pathway. Knockdown of LINC00662 can effectively promote the apoptosis of docetaxel-resistant cells and reduce their metastatic ability (97). METTL3 mediates the overexpression of MALAT1 in adriamycin resistant breast cancer through m6A. Subsequently, MALAT1 activates AGR2 transcription by recruiting E2F1 to increase the resistance of breast cancer cells to adriamycin and promotes cell proliferation and metastasis. In addition, silencing AGR2 effectively reduced the size and weight of tumor suppressors (99). Overexpression of piR-17560 derived from senescent neutrophil exosomes conferred docetaxel resistance to tumor cells, mainly through piR-17560/FTO/ZEB1 axis. Subsequent studies confirmed that upregulated ZEB1 could significantly promote chemoresistance and EMT of BC cells (105). Wtap-induced DLGAP1-AS1 was overexpressed in ADR-resistant BC cells. Further studies found that DLGAP1-AS1 promoted its expression in a positive feedback manner by targeting miR-299-3p/WTAP axis and increased the drug resistance of BC cells by promoting their proliferation and inhibiting apoptosis (107). CUL4B increased the expression of ER-α36 at the post-transcriptional level by inhibiting miR-32-5p, which reduced the sensitivity of breast cancer cells to tamoxifen. Moreover, knockdown of CUL4B effectively inhibited the growth of tamoxifen-resistant tumor grafts (110). UCA1 was upregulated in tamoxifen-resistant cells and was able to significantly enhance the viability of cells treated with tamoxifen. By silencing the expression of UCA1, it was able to promote G2/M phase cell cycle arrest, which was achieved by reducing the expression of p21 by UCA1. In addition, UCA1 significantly enhanced the resistance of breast cancer cells to tamoxifen by activating CAMP responsive element binding protein (CREB) through PI3K/AkT-dependent pathway (112). miR-22 plays a dual role in fulvestrant-resistant BC cells. Knockdown of miR-22 decreased the expression of cyclin E, up-regulated the expression of p21Cip1/Waf1 and p27Kip1, significantly inhibited cell proliferation, induced cell apoptosis and caused cell cycle arrest in S phase. Surprisingly, overexpression of miR-22 plays the same biological role. This implies that knockdown or ectopic expression of miR-22 is able to induce S-phase arrest and apoptosis by up-regulating p21Cip1/Waf1 and p27Kip1, which re-sensitizing drug-resistant cells to fulvestrant (119).

## Conclusion

As one of the forms of epigenetic modification, ncRNA has been intensively studied in the occurrence and development of breast cancer. At the same time, with the development of epitranscriptomics, further studies have found that ncRNAs can interact with other epigenetic modifications, such as DNA methylation, RNA methylation and histone modification, through different pathways. However, at present, the biological role of ncRNA and RNA methylation in breast cancer mainly focuses on m6A modification, and others such as m5C modification and m7G modification have not been confirmed. The ubiquitination modification and hematoxylin modification in histone modification also need to be further revealed.

This review summarizes the mechanisms and biological implications of ncRNAs and other major epigenetic modifications in breast cancer, but there is still much work to be done to study the dysregulation of epigenetic mechanisms related to pathological processes. Further studies may focus on less studied RNA modifications and histone modifications, such as m1A modification, m5C modification, etc. In addition, additional clinical trials are needed to determine the potential diagnostic and therapeutic effects of ncRNAs interacting with other epigenetic modifications in breast cancer patients.

## Author contributions

XP: Writing – review & editing. JX: Investigation, Visualization, Writing – original draft. LG: Writing – original draft. BX: Writing – review & editing. XW: Writing – review & editing.

## References

[1] Trayes KP Cokenakes SEH. Breast cancer treatment. Am Fam Physician (2021) 104(2):171–8. 34383430

[2] Kashyap D Pal D Sharma R Garg VK Goel N Koundal D. Global increase in breast cancer incidence: risk factors and preventive measures. BioMed Res Int (2022) 2022:9605439. doi: 10.1155/2022/9605439 35480139 PMC9038417

[3] Wang X Wang C Guan J Chen B Xu L Chen C. Progress of Breast Cancer basic research in China. Int J Biol Sci (2021) 17(8):2069–79. doi: 10.7150/ijbs.60631 PMC8193257 34131406

[4] Li Y Zhang H Merkher Y Chen L Liu N Leonov S. Recent advances in therapeutic strategies for triple-negative breast cancer. J Hematol Oncol (2022) 15(1):121. doi: 10.1186/s13045-022-01341-0 36038913 PMC9422136

[5] Zeng X Liu C Yao J Wan H Wan G Li Y. Breast cancer stem cells, heterogeneity, targeting therapies and therapeutic implications. Pharmacol Res (2021) 163:105320. doi: 10.1016/j.phrs.2020.105320 33271295

[6] Lv W Ren Y Hou K Hu W Yi Y Xiong M. Epigenetic modification mechanisms involved in keloid: current status and prospect. Clin Epigenet (2020) 12(1):183. doi: 10.1186/s13148-020-00981-8 PMC7690154 33243301

[7] Gjaltema RAF Rots MG. Advances of epigenetic editing. Curr Opin Chem Biol (2020) 57:75–81. doi: 10.1016/j.cbpa.2020.04.020 32619853

[8] Saghafinia S Mina M Riggi N Hanahan D Ciriello G. Pan-cancer landscape of aberrant DNA methylation across human tumors. Cell Rep (2018) 25(4):1066–1080.e8. doi: 10.1016/j.celrep.2018.09.082 30355485

[9] Nishiyama A Nakanishi M. Navigating the DNA methylation landscape of cancer. Trends Genet (2021) 37(11):1012–27. doi: 10.1016/j.tig.2021.05.002 34120771

[10] Papanicolau-Sengos A Aldape K. DNA methylation profiling: an emerging paradigm for cancer diagnosis. Annu Rev Pathol (2022) 17:295–321. doi: 10.1146/annurev-pathol-042220-022304 34736341

[11] Bajbouj K Al-Ali A Ramakrishnan RK Saber-Ayad M Hamid Q. Histone modification in NSCLC: molecular mechanisms and therapeutic targets. Int J Mol Sci (2021) 22(21):11701. doi: 10.3390/ijms222111701 34769131 PMC8584007

[12] Millán-Zambrano G Burton A Bannister AJ Schneider R. Histone post-translational modifications - cause and consequence of genome function. Nat Rev Genet (2022) 23(9):563–80. doi: 10.1038/s41576-022-00468-7 35338361

[13] Andrés M García-Gomis D Ponte I Suau P Roque A. Histone H1 post-translational modifications: update and future perspectives. Int J Mol Sci (2020) 21(16):5941. doi: 10.3390/ijms21165941 32824860 PMC7460583

[14] Li Y Chen X Lu C. The interplay between DNA and histone methylation: molecular mechanisms and disease implications. EMBO Rep (2021) 22(5):e51803. doi: 10.15252/embr.202051803 33844406 PMC8097341

[15] Song P Tayier S Cai Z Jia G. RNA methylation in mammalian development and cancer. Cell Biol Toxicol (2021) 37(6):811–31. doi: 10.1007/s10565-021-09627-8 PMC8599391 34272618

[16] He PC He C. m(6) A RNA methylation: from mechanisms to therapeutic potential. EMBO J (2021) 40(3):e105977. doi: 10.15252/embj.2020105977 33470439 PMC7849164

[17] Li W Hao Y Zhang X Xu S Pang D. Targeting RNA N(6)-methyladenosine modification: a precise weapon in overcoming tumor immune escape. Mol Cancer (2022) 21(1):176. doi: 10.1186/s12943-022-01652-3 36071523 PMC9454167

[18] Yan H Bu P. Non-coding RNA in cancer. Essays Biochem (2021) 65(4):625–39. doi: 10.1042/ebc20200032 PMC8564738 33860799

[19] Xue C Gu X Bao Z Su Y Lu J Li L. The mechanism underlying the ncRNA dysregulation pattern in hepatocellular carcinoma and its tumor microenvironment. Front Immunol (2022) 13:847728. doi: 10.3389/fimmu.2022.847728 35281015 PMC8904560

[20] Yang Q Chen Y Guo R Dai Y Tang L Zhao Y. Interaction of ncRNA and epigenetic modifications in gastric cancer: focus on histone modification. Front Oncol (2021) 11:822745. doi: 10.3389/fonc.2021.822745 35155211 PMC8826423

[21] Nagaraju GP Dariya B Kasa P Peela S El-Rayes BF. Epigenetics in hepatocellular carcinoma. Semin Cancer Biol 86(Pt (2022) 3):622–32. doi: 10.1016/j.semcancer.2021.07.017 34324953

[22] Zhang L Lu Q Chang C. Epigenetics in health and disease. Adv Exp Med Biol (2020) 1253:3–55. doi: 10.1007/978-981-15-3449-2_1 32445090

[23] Woo V Alenghat T. Epigenetic regulation by gut microbiota. Gut Microbes (2022) 14(1):2022407. doi: 10.1080/19490976.2021.2022407 35000562 PMC8744890

[24] Teng PC Liang Y Yarmishyn AA Hsiao YJ Lin TY Lin TW. RNA modifications and epigenetics in modulation of lung cancer and pulmonary diseases. Int J Mol Sci (2021) 22(19):10592. doi: 10.3390/ijms221910592 34638933 PMC8508636

[25] Fernández-Barrena MG Arechederra M Colyn L Berasain C Avila MA. Epigenetics in hepatocellular carcinoma development and therapy: The tip of the iceberg. JHEP Rep (2020) 2(6):100167. doi: 10.1016/j.jhepr.2020.100167 33134907 PMC7585149

[26] Mattei AL Bailly N Meissner A. DNA methylation: a historical perspective. Trends Genet (2022) 38(7):676–707. doi: 10.1016/j.tig.2022.03.010 35504755

[27] Ma S Chen C Ji X Liu J Zhou Q Wang G. The interplay between m6A RNA methylation and noncoding RNA in cancer. J Hematol Oncol (2019) 12(1):121. doi: 10.1186/s13045-019-0805-7 31757221 PMC6874823

[28] Hyun K Jeon J Park K Kim J. Writing, erasing and reading histone lysine methylations. Exp Mol Med (2017) 49(4):e324. doi: 10.1038/emm.2017.11 28450737 PMC6130214

[29] Edwards JR Yarychkivska O Boulard M Bestor TH. DNA methylation and DNA methyltransferases. Epigenet Chromatin (2017) 10:23. doi: 10.1186/s13072-017-0130-8 PMC5422929 28503201

[30] Moore LD Le T Fan G. DNA methylation and its basic function. Neuropsychopharmacology (2013) 38(1):23–38. doi: 10.1038/npp.2012.112 22781841 PMC3521964

[31] Meng H Cao Y Qin J Song X Zhang Q Shi Y. DNA methylation, its mediators and genome integrity. Int J Biol Sci (2015) 11(5):604–17. doi: 10.7150/ijbs.11218 PMC4400391 25892967

[32] Law PP Holland ML. DNA methylation at the crossroads of gene and environment interactions. Essays Biochem (2019) 63(6):717–26. doi: 10.1042/ebc20190031 PMC6923319 31782496

[33] Angeloni A Bogdanovic O. Enhancer DNA methylation: implications for gene regulation. Essays Biochem (2019) 63(6):707–15. doi: 10.1042/ebc20190030 31551326

[34] Chen Z Zhang Y. Role of mammalian DNA methyltransferases in development. Annu Rev Biochem (2020) 89:135–58. doi: 10.1146/annurev-biochem-103019-102815 31815535

[35] Wong KK. DNMT1: A key drug target in triple-negative breast cancer. Semin Cancer Biol (2021) 72:198–213. doi: 10.1016/j.semcancer.2020.05.010 32461152

[36] Zhang ZM Lu R Wang P Yu Y Chen D Gao L. Structural basis for DNMT3A-mediated *de novo* DNA methylation. Nature (2018) 554(7692):387–91. doi: 10.1038/nature25477 PMC5814352 29414941

[37] Man X Li Q Wang B Zhang H Zhang S Li Z. DNMT3A and DNMT3B in breast tumorigenesis and potential therapy. Front Cell Dev Biol (2022) 10:916725. doi: 10.3389/fcell.2022.916725 35620052 PMC9127442

[38] Zhang M Song J Yuan W Zhang W Sun Z. Roles of RNA methylation on tumor immunity and clinical implications. Front Immunol (2021) 12:641507. doi: 10.3389/fimmu.2021.641507 33777035 PMC7987906

[39] Wu Y Wang Z Han L Guo Z Yan B Guo L. PRMT5 regulates RNA m6A demethylation for doxorubicin sensitivity in breast cancer. Mol Ther (2022) 30(7):2603–17. doi: 10.1016/j.ymthe.2022.03.003 PMC9263239 35278676

[40] Zhou M Dong M Yang X Gong J Liao X Zhang Q. The emerging roles and mechanism of m6a in breast cancer progression. Front Genet (2022) 13:983564. doi: 10.3389/fgene.2022.983564 36035182 PMC9399344

[41] Chen M Wong CM. The emerging roles of N6-methyladenosine (m6A) deregulation in liver carcinogenesis. Mol Cancer (2020) 19(1):44. doi: 10.1186/s12943-020-01172-y 32111216 PMC7047367

[42] Oerum S Meynier V Catala M Tisné C. A comprehensive review of m6A/m6Am RNA methyltransferase structures. Nucleic Acids Res (2021) 49(13):7239–55. doi: 10.1093/nar/gkab378 PMC8287941 34023900

[43] Wang T Kong S Tao M Ju S. The potential role of RNA N6-methyladenosine in Cancer progression. Mol Cancer (2020) 19(1):88. doi: 10.1186/s12943-020-01204-7 32398132 PMC7216508

[44] Zeng C Huang W Li Y Weng H. Roles of METTL3 in cancer: mechanisms and therapeutic targeting. J Hematol Oncol (2020) 13(1):117. doi: 10.1186/s13045-020-00951-w 32854717 PMC7457244

[45] Zhou H Yin K Zhang Y Tian J Wang S. The RNA m6A writer METTL14 in cancers: Roles, structures, and applications. Biochim Biophys Acta Rev Cancer (2021) 1876(2):188609. doi: 10.1016/j.bbcan.2021.188609 34375716

[46] Chen J Fang Y Xu Y Sun H. Role of m6A modification in female infertility and reproductive system diseases. Int J Biol Sci (2022) 18(9):3592–604. doi: 10.7150/ijbs.69771 PMC9254474 35813486

[47] Zhang B Jiang H Dong Z Sun A Ge J. The critical roles of m6A modification in metabolic abnormality and cardiovascular diseases. Genes Dis (2021) 8(6):746–58. doi: 10.1016/j.gendis.2020.07.011 PMC8427257 34522705

[48] Wang J Wang J Gu Q Ma Y Yang Y Zhu J. The biological function of m6A demethylase ALKBH5 and its role in human disease. Cancer Cell Int (2020) 20:347. doi: 10.1186/s12935-020-01450-1 32742194 PMC7388453

[49] Fang Z Mei W Qu C Lu J Shang L Cao F. Role of m6A writers, erasers and readers in cancer. Exp Hematol Oncol (2022) 11(1):45. doi: 10.1186/s40164-022-00298-7 35945641 PMC9361621

[50] Bannister AJ Kouzarides T. Regulation of chromatin by histone modifications. Cell Res (2011) 21(3):381–95. doi: 10.1038/cr.2011.22 PMC3193420 21321607

[51] Lawrence M Daujat S Schneider R. Lateral thinking: how histone modifications regulate gene expression. Trends Genet (2016) 32(1):42–56. doi: 10.1016/j.tig.2015.10.007 26704082

[52] He W Li Q Li X. Acetyl-CoA regulates lipid metabolism and histone acetylation modification in cancer. Biochim Biophys Acta Rev Cancer (2023) 1878(1):188837. doi: 10.1016/j.bbcan.2022.188837 36403921

[53] Zaib S Rana N Khan I. Histone modifications and their role in epigenetics of cancer. Curr Med Chem (2022) 29(14):2399–411. doi: 10.2174/0929867328666211108105214 34749606

[54] Lin Y Qiu T Wei G Que Y Wang W Kong Y. Role of histone post-translational modifications in inflammatory diseases. Front Immunol (2022) 13:852272. doi: 10.3389/fimmu.2022.852272 35280995 PMC8908311

[55] Ito T. Role of histone modification in chromatin dynamics. J Biochem (2007) 141(5):609–14. doi: 10.1093/jb/mvm091 17405795

[56] Portela A Esteller M. Epigenetic modifications and human disease. Nat Biotechnol (2010) 28(10):1057–68. doi: 10.1038/nbt.1685 20944598

[57] Collins BE Greer CB Coleman BC Sweatt JD. Histone H3 lysine K4 methylation and its role in learning and memory. Epigenet Chromatin (2019) 12(1):7. doi: 10.1186/s13072-018-0251-8 PMC6322263 30616667

[58] Shvedunova M Akhtar A. Modulation of cellular processes by histone and non-histone protein acetylation. Nat Rev Mol Cell Biol (2022) 23(5):329–49. doi: 10.1038/s41580-021-00441-y 35042977

[59] An S Camarillo JM Huang TY Li D Morris JA Zoltek MA. Histone tail analysis reveals H3K36me2 and H4K16ac as epigenetic signatures of diffuse intrinsic pontine glioma. J Exp Clin Cancer Res (2020) 39(1):261. doi: 10.1186/s13046-020-01773-x 33239043 PMC7687710

[60] Seto E Yoshida M. Erasers of histone acetylation: the histone deacetylase enzymes. Cold Spring Harb Perspect Biol (2014) 6(4):a018713. doi: 10.1101/cshperspect.a018713 24691964 PMC3970420

[61] Komar D Juszczynski P. Rebelled epigenome: histone H3S10 phosphorylation and H3S10 kinases in cancer biology and therapy. Clin Epigenet (2020) 12(1):147. doi: 10.1186/s13148-020-00941-2 PMC7556946 33054831

[62] Harshman SW Hoover ME Huang C Branson OE Chaney SB Cheney CM. Histone H1 phosphorylation in breast cancer. J Proteome Res (2014) 13(5):2453–67. doi: 10.1021/pr401248f PMC4012839 24601643

[63] Anastasiadou E Jacob LS Slack FJ. Non-coding RNA networks in cancer. Nat Rev Cancer (2018) 18(1):5–18. doi: 10.1038/nrc.2017.99 29170536 PMC6337726

[64] Xu J Wu KJ Jia QJ Ding XF. Roles of miRNA and lncRNA in triple-negative breast cancer. J Zhejiang Univ Sci B (2020) 21(9):673–89. doi: 10.1631/jzus.B1900709 PMC7519626 32893525

[65] Shi J Zhou T Chen Q. Exploring the expanding universe of small RNAs. Nat Cell Biol (2022) 24(4):415–23. doi: 10.1038/s41556-022-00880-5 PMC9035129 35414016

[66] Xiao L Wang J Ju S Cui M Jing R. Disorders and roles of tsRNA, snoRNA, snRNA and piRNA in cancer. J Med Genet (2022) 59(7):623–31. doi: 10.1136/jmedgenet-2021-108327 35145038

[67] Bridges MC Daulagala AC Kourtidis A. LNCcation: lncRNA localization and function. J Cell Biol (2021) 220(2):e202009045. doi: 10.1083/jcb.202009045 33464299 PMC7816648

[68] Zhang P Dai M. CircRNA: a rising star in plant biology. J Genet Genomics (2022) 49(12):1081–92. doi: 10.1016/j.jgg.2022.05.004 35644325

[69] Xue C Chu Q Zheng Q Jiang S Bao Z Su Y. Role of main RNA modifications in cancer: N(6)-methyladenosine, 5-methylcytosine, and pseudouridine. Signal Transduct Target Ther (2022) 7(1):142. doi: 10.1038/s41392-022-01003-0 35484099 PMC9051163

[70] Uddin MS Mamun AA Alghamdi BS Tewari D Jeandet P Sarwar MS. Epigenetics of glioblastoma multiforme: From molecular mechanisms to therapeutic approaches. Semin Cancer Biol (2022) 83:100–20. doi: 10.1016/j.semcancer.2020.12.015 33370605

[71] Lu J Huang Y Zhang X Xu Y Nie S. Noncoding RNAs involved in DNA methylation and histone methylation, and acetylation in diabetic vascular complications. Pharmacol Res (2021) 170:105520. doi: 10.1016/j.phrs.2021.105520 33639232

[72] Wang Q Liu J You Z Yin Y Liu L Kang Y. LncRNA TINCR favors tumorigenesis via STAT3-TINCR-EGFR-feedback loop by recruiting DNMT1 and acting as a competing endogenous RNA in human breast cancer. Cell Death Dis (2021) 12(1):83. doi: 10.1038/s41419-020-03188-0 33446634 PMC7809450

[73] Wang Q Li G Ma X Liu L Liu J Yin Y. LncRNA TINCR impairs the efficacy of immunotherapy against breast cancer by recruiting DNMT1 and downregulating MiR-199a-5p via the STAT1-TINCR-USP20-PD-L1 axis. Cell Death Dis (2023) 14(2):76. doi: 10.1038/s41419-023-05609-2 36725842 PMC9892521

[74] Shen B Li Y Ye Q Qin Y. YY1-mediated long non-coding RNA Kcnq1ot1 promotes the tumor progression by regulating PTEN via DNMT1 in triple negative breast cancer. Cancer Gene Ther (2021) 28(10-11):1099–112. doi: 10.1038/s41417-020-00254-9 33323961

[75] Pan T Ding H Jin L Zhang S Wu D Pan W. DNMT1-mediated demethylation of lncRNA MEG3 promoter suppressed breast cancer progression by repressing Notch1 signaling pathway. Cell Cycle (2022) 21(21):2323–37. doi: 10.1080/15384101.2022.2094662 PMC9586644 35822955

[76] Fu Y Zhang X Liu X Wang P Chu W Zhao W. The DNMT1-PAS1-PH20 axis drives breast cancer growth and metastasis. Signal Transduct Target Ther (2022) 7(1):81. doi: 10.1038/s41392-022-00896-1 35307730 PMC8934873

[77] Li H Li HH Chen Q Wang YY Fan CC Duan YY. miR-142-5p inhibits cell invasion and migration by targeting DNMT1 in breast cancer. Oncol Res (2022) 28(9):885–97. doi: 10.3727/096504021x16274672547967 PMC8790130 34321149

[78] Liu T Wang J Sun L Li M He X Jiang J. Piwi-interacting RNA-651 promotes cell proliferation and migration and inhibits apoptosis in breast cancer by facilitating DNMT1-mediated PTEN promoter methylation. Cell Cycle (2021) 20(16):1603–16. doi: 10.1080/15384101.2021.1956090 PMC8409782 34313525

[79] Li Y Jiang B He Z Zhu H He R Fan S. circIQCH sponges miR-145 to promote breast cancer progression by upregulating DNMT3A expression. Aging (Albany NY) (2020) 12(15):15532–45. doi: 10.18632/aging.103746 PMC7467367 32756009

[80] Noyan S Andac Ozketen A Gurdal H Gur Dedeoglu B. miR-770-5p regulates EMT and invasion in TNBC cells by targeting DNMT3A. Cell Signal (2021) 83:109996. doi: 10.1016/j.cellsig.2021.109996 33798630

[81] Wang J Xie S Yang J Xiong H Jia Y Zhou Y. The long noncoding RNA H19 promotes tamoxifen resistance in breast cancer via autophagy. J Hematol Oncol (2019) 12(1):81. doi: 10.1186/s13045-019-0747-0 31340867 PMC6657081

[82] Huang ZF Tang YL Shen ZL Yang KY Gao K. UXT, a novel DNMT3b-binding protein, promotes breast cancer progression via negatively modulating lncRNA MEG3/p53 axis. Mol Ther Oncolytics (2022) 24:497–506. doi: 10.1016/j.omto.2021.12.008 35229028 PMC8850569

[83] Li H Wang P Liu J Liu W Wu X Ding J. Hypermethylation of lncRNA MEG3 impairs chemosensitivity of breast cancer cells. J Clin Lab Anal (2020) 34(9):e23369. doi: 10.1002/jcla.23369 32618397 PMC7521317

[84] Li L Gan YP Peng H. RAMP2-AS1 inhibits CXCL11 expression to suppress Malignant phenotype of breast cancer by recruiting DNMT1 and DNMT3B. Exp Cell Res (2022) 416(2):113139. doi: 10.1016/j.yexcr.2022.113139 35390315

[85] Li S Hu J Li G Mai H Gao Y Liang B. Epigenetic regulation of LINC01270 in breast cancer progression by mediating LAMA2 promoter methylation and MAPK signaling pathway. Cell Biol Toxicol (2022) 39(4):1359–75. doi: 10.1007/s10565-022-09763-9 36241925

[86] Li F Yuan P Rao M Jin CH Tang W Rong YF. piRNA-independent function of PIWIL1 as a co-activator for anaphase promoting complex/cyclosome to drive pancreatic cancer metastasis. Nat Cell Biol (2020) 22(4):425–38. doi: 10.1038/s41556-020-0486-z 32203416

[87] Wang Y Dong T Wang P Li S Wu G Zhou J. LINC00922 regulates epithelial-mesenchymal transition, invasive and migratory capacities in breast cancer through promoting NKD2 methylation. Cell Signal (2021) 77:109808. doi: 10.1016/j.cellsig.2020.109808 33045317

[88] De Blasio A Di Fiore R Pratelli G Drago-Ferrante R Saliba C Baldacchino S. A loop involving NRF2, miR-29b-1-5p and AKT, regulates cell fate of MDA-MB-231 triple-negative breast cancer cells. J Cell Physiol (2020) 235(2):629–37. doi: 10.1002/jcp.29062 31313842

[89] Shao G Fan X Zhang P Liu X Huang L Ji S. Methylation-dependent MCM6 repression induced by LINC00472 inhibits triple-negative breast cancer metastasis by disturbing the MEK/ERK signaling pathway. Aging (Albany NY) (2021) 13(4):4962–75. doi: 10.18632/aging.103568 PMC7950301 33668040

[90] Wang HB Wei H Wang JS Li L Chen AY Li ZG. Down-regulated expression of LINC00518 prevents epithelial cell growth and metastasis in breast cancer through the inhibition of CDX2 methylation and the Wnt signaling pathway. Biochim Biophys Acta Mol Basis Dis (2019) 1865(3):708–23. doi: 10.1016/j.bbadis.2019.01.003 30611858

[91] Xu X Yuan X Ni J Guo J Gao Y Yin W. MAGI2-AS3 inhibits breast cancer by downregulating DNA methylation of MAGI2. J Cell Physiol (2021) 236(2):1116–30. doi: 10.1002/jcp.29922 32730644

[92] Tian Y Chen ZH Wu P Zhang D Ma Y Liu XF. MIR497HG-Derived miR-195 and miR-497 Mediate Tamoxifen Resistance via PI3K/AKT Signaling in Breast Cancer. Adv Sci (Weinh) (2023) 10(12):e2204819. doi: 10.1002/advs.202204819 36815359 PMC10131819

[93] Ruan HG Gu WC Xia W Gong Y Zhou XL Chen WY. METTL3 is suppressed by circular RNA circMETTL3/miR-34c-3p signaling and limits the tumor growth and metastasis in triple negative breast cancer. Front Oncol (2021) 11:778132. doi: 10.3389/fonc.2021.778132 35004298 PMC8727604

[94] Rong D Dong Q Qu H Deng X Gao F Li Q. m(6)A-induced LINC00958 promotes breast cancer tumorigenesis via the miR-378a-3p/YY1 axis. Cell Death Discovery (2021) 7(1):27. doi: 10.1038/s41420-020-00382-z 33531456 PMC7854648

[95] Guo Y Feng L. N6-methyladenosine-mediated upregulation of LINC00520 accelerates breast cancer progression via regulating miR-577/POSTN axis and downstream ILK/AKT/mTOR signaling pathway. Arch Biochem Biophys (2022) 729:109381. doi: 10.1016/j.abb.2022.109381 36027936

[96] Fan S Wang L. N(6)-Methyladenosine-regulated LINC00675 suppress the proliferation, migration and invasion of breast cancer cells via inhibiting miR-513b-5p. Bioengineered (2021) 12(2):10690–702. doi: 10.1080/21655979.2021.2001905 PMC8810037 34738869

[97] Jing L Lan L Mingxin Z Zhaofeng Z. METTL3/LINC00662/miR-186-5p feedback loop regulates docetaxel resistance in triple negative breast cancer. Sci Rep (2022) 12(1):16715. doi: 10.1038/s41598-022-20477-0 36202872 PMC9537189

[98] Zhao C Ling X Xia Y Yan B Guan Q. The m6A methyltransferase METTL3 controls epithelial-mesenchymal transition, migration and invasion of breast cancer through the MALAT1/miR-26b/HMGA2 axis. Cancer Cell Int (2021) 21(1):441. doi: 10.1186/s12935-021-02113-5 34419065 PMC8380348

[99] Li S Jiang F Chen F Deng Y Pan X. Effect of m6A methyltransferase METTL3 -mediated MALAT1/E2F1/AGR2 axis on adriamycin resistance in breast cancer. J Biochem Mol Toxicol (2022) 36(1):e22922. doi: 10.1002/jbt.22922 34964205

[100] Xu LM Zhang J Ma Y Yuan YJ Yu H Wang J. MicroRNA-135 inhibits initiation of epithelial-mesenchymal transition in breast cancer by targeting ZNF217 and promoting m6A modification of NANOG. Oncogene (2022) 41(12):1742–51. doi: 10.1038/s41388-022-02211-2 35121826

[101] Sun T Wu Z Wang X Wang Y Hu X Qin W. LNC942 promoting METTL14-mediated m(6)A methylation in breast cancer cell proliferation and progression. Oncogene (2020) 39(31):5358–72. doi: 10.1038/s41388-020-1338-9 32576970

[102] Zhao C Ling X Xia Y Yan B Guan Q. LncRNA UCA1 promotes SOX12 expression in breast cancer by regulating m(6)A modification of miR-375 by METTL14 through DNA methylation. Cancer Gene Ther (2022) 29(7):1043–55. doi: 10.1038/s41417-021-00390-w 35022519

[103] Yi D Wang R Shi X Xu L Yilihamu Y Sang J. METTL14 promotes the migration and invasion of breast cancer cells by modulating N6−methyladenosine and hsa−miR−146a−5p expression. Oncol Rep (2020) 43(5):1375–86. doi: 10.3892/or.2020.7515 PMC7107905 32323801

[104] Xu Y Ye S Zhang N Zheng S Liu H Zhou K. The FTO/miR-181b-3p/ARL5B signaling pathway regulates cell migration and invasion in breast cancer. Cancer Commun (Lond) (2020) 40(10):484–500. doi: 10.1002/cac2.12075 32805088 PMC7571404

[105] Ou B Liu Y Gao Z Xu J Yan Y Li Y. Senescent neutrophils-derived exosomal piRNA-17560 promotes chemoresistance and EMT of breast cancer via FTO-mediated m6A demethylation. Cell Death Dis (2022) 13(10):905. doi: 10.1038/s41419-022-05317-3 36302751 PMC9613690

[106] Zhang M Wang J Jin Y Zheng Q Xing M Tang Y. YTHDF2-mediated FGF14-AS2 decay promotes osteolytic metastasis of breast cancer by enhancing RUNX2 mRNA translation. Br J Cancer (2022) 127(12):2141–53. doi: 10.1038/s41416-022-02006-y PMC9726880 36216883

[107] Huang T Cao L Feng N Xu B Dong Y Wang M. N(6)-methyladenosine (m(6)A)-mediated lncRNA DLGAP1-AS1enhances breast canceradriamycin resistance through miR-299-3p/WTAP feedback loop. Bioengineered (2021) 12(2):10935–44. doi: 10.1080/21655979.2021.2000198 PMC8809972 34866525

[108] Wu Y Shi W Tang T Wang Y Yin X Chen Y. miR-29a contributes to breast cancer cells epithelial-mesenchymal transition, migration, and invasion via down-regulating histone H4K20 trimethylation through directly targeting SUV420H2. Cell Death Dis (2019) 10(3):176. doi: 10.1038/s41419-019-1437-0 30792382 PMC6385178

[109] Zhang M Yang L Hou L Tang X. LncRNA SNHG1 promotes tumor progression and cisplatin resistance through epigenetically silencing miR-381 in breast cancer. Bioengineered (2021) 12(2):9239–50. doi: 10.1080/21655979.2021.1996305 PMC8809974 34806925

[110] Wang Y Pan X Li Y Wang R Yang Y Jiang B. CUL4B renders breast cancer cells tamoxifen-resistant via miR-32-5p/ER-α36 axis. J Pathol (2021) 254(2):185–98. doi: 10.1002/path.5657 33638154

[111] Jin ML Kim YW Jin HL Kang H Lee EK Stallcup MR. Aberrant expression of SETD1A promotes survival and migration of estrogen receptor α-positive breast cancer cells. Int J Cancer (2018) 143(11):2871–83. doi: 10.1002/ijc.31853 PMC6278950 30191958

[112] Li Z Yu D Li H Lv Y Li S. Long non−coding RNA UCA1 confers tamoxifen resistance in breast cancer endocrinotherapy through regulation of the EZH2/p21 axis and the PI3K/AKT signaling pathway. Int J Oncol (2019) 54(3):1033–42. doi: 10.3892/ijo.2019.4679 30628639

[113] Xun J Du L Gao R Shen L Wang D Kang L. Cancer-derived exosomal miR-138-5p modulates polarization of tumor-associated macrophages through inhibition of KDM6B. Theranostics (2021) 11(14):6847–59. doi: 10.7150/thno.51864 PMC8171095 34093857

[114] Hu A Hong F Li D Jin Y Kon L Xu Z. Long non-coding RNA ROR recruits histone transmethylase MLL1 to up-regulate TIMP3 expression and promote breast cancer progression. J Transl Med (2021) 19(1):95. doi: 10.1186/s12967-020-02682-5 33653378 PMC7927245

[115] Lu C Wang X Zhao X Xin Y Liu C. Long non-coding RNA ARAP1-AS1 accelerates cell proliferation and migration in breast cancer through miR-2110/HDAC2/PLIN1 axis. Biosci Rep (2020) 40(4):BSR20191764. doi: 10.1042/bsr20191764 32110804 PMC7197975

[116] Rahbari R Rahimi K Rasmi Y Khadem-Ansari MH Abdi M. miR-589-5p inhibits cell proliferation by targeting histone deacetylase 3 in triple negative breast cancer. Arch Med Res (2022) 53(5):483–91. doi: 10.1016/j.arcmed.2022.06.006 35840467

[117] Chen Z Pei L Zhang D Xu F Zhou E Chen X. HDAC3 increases HMGB3 expression to facilitate the immune escape of breast cancer cells via down-regulating microRNA-130a-3p. Int J Biochem Cell Biol (2021) 135:105967. doi: 10.1016/j.biocel.2021.105967 33727043

[118] Menbari MN Rahimi K Ahmadi A Elyasi A Darvishi N Hosseini V. MiR-216b-5p inhibits cell proliferation in human breast cancer by down-regulating HDAC8 expression. Life Sci (2019) 237:116945. doi: 10.1016/j.lfs.2019.116945 31605710

[119] Wang B Li D Filkowski J Rodriguez-Juarez R Storozynsky Q Malach M. A dual role of miR-22 modulated by RelA/p65 in resensitizing fulvestrant-resistant breast cancer cells to fulvestrant by targeting FOXP1 and HDAC4 and constitutive acetylation of p53 at Lys382. Oncogenesis (2018) 7(7):54. doi: 10.1038/s41389-018-0063-5 30057418 PMC6064715

[120] Shi X Wang X. LINC00473 mediates cyclin D1 expression through a balance between activation and repression signals in breast cancer cells. FEBS Lett (2019) 593(7):751–9. doi: 10.1002/1873-3468.13353 30848493

[121] Zhou L Li H Sun T Wen X Niu C Li M. HULC targets the IGF1R-PI3K-AKT axis in trans to promote breast cancer metastasis and cisplatin resistance. Cancer Lett (2022) 548:215861. doi: 10.1016/j.canlet.2022.215861 35981570

[122] Pang B Sui S Wang Q Wu J Yin Y Xu S. Upregulation of DLEU1 expression by epigenetic modification promotes tumorigenesis in human cancer. J Cell Physiol (2019) 234(10):17420–32. doi: 10.1002/jcp.28364 30793303

[123] Dong H Wang W Mo S Liu Q Chen X Chen R. Long non-coding RNA SNHG14 induces trastuzumab resistance of breast cancer via regulating PABPC1 expression through H3K27 acetylation. J Cell Mol Med (2018) 22(10):4935–47. doi: 10.1111/jcmm.13758 PMC6156344 30063126

[124] Xiong Y Gu Y Wang F Li L Zhu M Wang N. LINC01857 as an oncogene regulates CREB1 activation by interacting with CREBBP in breast cancer. J Cell Physiol (2019) 234(8):14031–9. doi: 10.1002/jcp.28090 PMC13483081 30628071

[125] Abdolvahabi Z Nourbakhsh M Hosseinkhani S Hesari Z Alipour M Jafarzadeh M. MicroRNA-590-3P suppresses cell survival and triggers breast cancer cell apoptosis via targeting sirtuin-1 and deacetylation of p53. J Cell Biochem (2019) 120(6):9356–68. doi: 10.1002/jcb.28211 30520099

[126] Fan SJ Cui Y Li YH Xu JC Shen YY Huang H. LncRNA CASC9 activated by STAT3 promotes the invasion of breast cancer and the formation of lymphatic vessels by enhancing H3K27ac-activated SOX4. Kaohsiung J Med Sci (2022) 38(9):848–57. doi: 10.1002/kjm2.12573 PMC11896238 35860965

[127] Xie N Zhang R Bi Z Ren W You K Hu H. H3K27 acetylation activated long noncoding RNA RP11-162G10.5 promotes breast cancer progression via the YBX1/GLO1 axis. Cell Oncol (Dordr) (2023) 46(2):375–90. doi: 10.1007/s13402-022-00756-8 PMC12974685 36576700

[128] Surapaneni SK Bhat ZR Tikoo K. MicroRNA-941 regulates the proliferation of breast cancer cells by altering histone H3 Ser 10 phosphorylation. Sci Rep (2020) 10(1):17954. doi: 10.1038/s41598-020-74847-7 33087811 PMC7578795

[129] Okugawa Y Grady WM Goel A. Epigenetic alterations in colorectal cancer: emerging biomarkers. Gastroenterology (2015) 149(5):1204–1225.e12. doi: 10.1053/j.gastro.2015.07.011 26216839 PMC4589488

[130] Han X Guo J Fan Z. Interactions between m6A modification and miRNAs in Malignant tumors. Cell Death Dis (2021) 12(6):598. doi: 10.1038/s41419-021-03868-5 34108450 PMC8190295

[131] Lin H Wang Y Wang P Long F Wang T. Mutual regulation between N6-methyladenosine (m6A) modification and circular RNAs in cancer: impacts on therapeutic resistance. Mol Cancer (2022) 21(1):148. doi: 10.1186/s12943-022-01620-x 35843942 PMC9290271

[132] Ren S Zhang Y Yang X Li X Zheng Y Liu Y. N6-methyladenine- induced LINC00667 promoted breast cancer progression through m6A/KIAA1429 positive feedback loop. Bioengineered (2022) 13(5):13462–73. doi: 10.1080/21655979.2022.2077893 PMC9275968 36700472

[133] Liu Y Dong Y He X Gong A Gao J Hao X. piR-hsa-211106 inhibits the progression of lung adenocarcinoma through pyruvate carboxylase and enhances chemotherapy sensitivity. Front Oncol (2021) 11:651915. doi: 10.3389/fonc.2021.651915 34249688 PMC8260943

[134] Pan X Hong X Li S Meng P Xiao F. METTL3 promotes adriamycin resistance in MCF-7 breast cancer cells by accelerating pri-microRNA-221-3p maturation in a m6A-dependent manner. Exp Mol Med (2021) 53(1):91–102. doi: 10.1038/s12276-020-00510-w 33420414 PMC8080609

[135] Slack FJ Chinnaiyan AM. The role of non-coding RNAs in oncology. Cell (2019) 179(5):1033–55. doi: 10.1016/j.cell.2019.10.017 PMC7347159 31730848

[136] McCabe EM Rasmussen TP. lncRNA involvement in cancer stem cell function and epithelial-mesenchymal transitions. Semin Cancer Biol (2021) 75:38–48. doi: 10.1016/j.semcancer.2020.12.012 33346133

[137] Liu S Sun Y Hou Y Yang L Wan X Qin Y. A novel lncRNA ROPM-mediated lipid metabolism governs breast cancer stem cell properties. J Hematol Oncol (2021) 14(1):178. doi: 10.1186/s13045-021-01194-z 34715882 PMC8555326

[138] Müller V Oliveira-Ferrer L Steinbach B Pantel K Schwarzenbach H. Interplay of lncRNA H19/miR-675 and lncRNA NEAT1/miR-204 in breast cancer. Mol Oncol (2019) 13(5):1137–49. doi: 10.1002/1878-0261.12472 PMC6487715 30803129

[139] Sun Z Shi K Yang S Liu J Zhou Q Wang G. Effect of exosomal miRNA on cancer biology and clinical applications. Mol Cancer (2018) 17(1):147. doi: 10.1186/s12943-018-0897-7 30309355 PMC6182840

[140] Sabit H Cevik E Tombuloglu H Abdel-Ghany S Tombuloglu G Esteller M. Triple negative breast cancer in the era of miRNA. Crit Rev Oncol Hematol (2021) 157:103196. doi: 10.1016/j.critrevonc.2020.103196 33307198

[141] Sengupta D Deb M Kar S Pradhan N Parbin S Kirtana R. Patra: Dissecting miRNA facilitated physiology and function in human breast cancer for therapeutic intervention. Semin Cancer Biol (2021) 72:46–64. doi: 10.1016/j.semcancer.2020.05.017 32497683

[142] Singh D Assaraf YG Gacche RN. Long non-coding RNA mediated drug resistance in breast cancer. Drug Resist Update (2022) 63:100851. doi: 10.1016/j.drup.2022.100851 35810716

[143] Hashemi M Arani HZ Orouei S Fallah S Ghorbani A Khaledabadi M. EMT mechanism in breast cancer metastasis and drug resistance: Revisiting molecular interactions and biological functions. BioMed Pharmacother (2022) 155:113774. doi: 10.1016/j.biopha.2022.113774 36271556

[144] Wen N Lv Q Du ZG. MicroRNAs involved in drug resistance of breast cancer by regulating autophagy. J Zhejiang Univ Sci B (2020) 21(9):690–702. doi: 10.1631/jzus.B2000076 32893526 PMC7519632

[145] Jamialahmadi K Zahedipour F Karimi G. The role of microRNAs on doxorubicin drug resistance in breast cancer. J Pharm Pharmacol (2021) 73(8):997–1006. doi: 10.1093/jpp/rgaa031 33942851

[146] Wang Z Wu X. Study and analysis of antitumor resistance mechanism of PD1/PD-L1 immune checkpoint blocker. Cancer Med (2020) 9(21):8086–121. doi: 10.1002/cam4.3410 PMC7643687 32875727

[147] Koual M Tomkiewicz C Cano-Sancho G Antignac JP Bats AS Coumoul X. Environmental chemicals, breast cancer progression and drug resistance. Environ Health (2020) 19(1):117. doi: 10.1186/s12940-020-00670-2 33203443 PMC7672852

[148] Garcia-Martinez L Zhang Y Nakata Y Chan HL Morey L. Epigenetic mechanisms in breast cancer therapy and resistance. Nat Commun (2021) 12(1):1786. doi: 10.1038/s41467-021-22024-3 33741974 PMC7979820

[149] Cocco S Leone A Roca MS Lombardi R Piezzo M Caputo R. Inhibition of autophagy by chloroquine prevents resistance to PI3K/AKT inhibitors and potentiates their antitumor effect in combination with paclitaxel in triple negative breast cancer models. J Transl Med (2022) 20(1):290. doi: 10.1186/s12967-022-03462-z 35761360 PMC9235112

[150] Zamame Ramirez JA Romagnoli GG Kaneno R. Inhibiting autophagy to prevent drug resistance and improve anti-tumor therapy. Life Sci (2021) 265:118745. doi: 10.1016/j.lfs.2020.118745 33186569

